# Analysis of factors affecting the variability of a quantitative suspension bead array assay measuring IgG to multiple *Plasmodium* antigens

**DOI:** 10.1371/journal.pone.0199278

**Published:** 2018-07-02

**Authors:** Itziar Ubillos, Ruth Aguilar, Hector Sanz, Alfons Jiménez, Marta Vidal, Aida Valmaseda, Yan Dong, Deepak Gaur, Chetan E. Chitnis, Sheetij Dutta, Evelina Angov, John J. Aponte, Joseph J. Campo, Clarissa Valim, Jaroslaw Harezlak, Carlota Dobaño

**Affiliations:** 1 ISGlobal, Hospital Clínic—Universitat de Barcelona, Barcelona, Catalonia, Spain; 2 CIBER Epidemiology and Public Health (CIBERESP), Barcelona, Spain; 3 Department of Biostatistics, RM Fairbanks School of Public Health, Indianapolis, IN, United States of America; 4 Malaria Group, International Centre for Genetic Engineering and Biotechnology (ICGEB), New Delhi, India; 5 Laboratory of Malaria and Vaccine Research, School of Biotechnology, Jawaharlal Nehru University, New Delhi, India; 6 U.S. Military Malaria Vaccine Program, Walter Reed Army Institute of Research (WRAIR), Silver Spring, Maryland, United States of America; 7 Department of Osteopathic Medical Specialties, Michigan State University, East Lansing, MI, United States of America; 8 Department of Immunology and Infectious Diseases, Harvard T.H. Chen School of Public Health, Boston, MA, United States of America; Ehime Daigaku, JAPAN

## Abstract

Reducing variability of quantitative suspension array assays is key for multi-center and large sero-epidemiological studies. To maximize precision and robustness of an *in-house* IgG multiplex assay, we analyzed the effect of several conditions on variability to find the best combination. The following assay conditions were studied through a fractional factorial design: antigen-bead coupling (stock vs. several), sample predilution (stock vs. daily), temperature of incubation of sample with antigen-bead (22°C vs. 37°C), plate washing (manual vs. automatic) and operator expertise (expert vs. apprentice). IgG levels against seven *P*. *falciparum* antigens with heterogeneous immunogenicities were measured in test samples, in a positive control and in blanks. We assessed the variability and MFI quantification range associated to each combination of conditions, and their interactions, and evaluated the minimum number of samples and blank replicates to achieve good replicability. Results showed that antigen immunogenicity and sample seroreactivity defined the optimal dilution to assess the effect of assay conditions on variability. We found that a unique antigen-bead coupling, samples prediluted daily, incubation at 22°C, and automatic washing, had lower variability. However, variability increased when performing several couplings and incubating at 22°C vs. 37°C. In addition, no effect of temperature was seen with a unique coupling. The expertise of the operator had no effect on assay variability but reduced the MFI quantification range. Finally, differences between sample replicates were minimal, and two blanks were sufficient to capture assay variability, as suggested by the constant Intraclass Correlation Coefficient of three and two blanks. To conclude, a single coupling was the variable that most consistently reduced assay variability, being clearly advisable. In addition, we suggest having more sample dilutions instead of replicates to increase the likelihood of sample MFIs falling in the linear part of the antigen-specific curve, thus increasing precision.

## Introduction

The identification of antibody biomarkers of antigen immunogenicity and protection against certain infectious diseases is particularly challenging when dealing with complex microbial pathogens like *Plasmodium falciparum*, with a proteome of over 5,000 proteins (www.plasmodb.org) and some polymorphic proteins [1]. In this context, sero-epidemiological studies and vaccine trials need to evaluate different immune responses with medium to high throughput standardized and robust assays. The enzyme-linked immuno-sorbent assay (ELISA) has been a widely used tool to evaluate biomarkers in life sciences research, clinical diagnostics, biosurveillance, and food safety. However, ELISA requires a relatively large amount of sample, and the surface area of the wells and potential hydrophobic binding of antibodies can lead to non-specific bindings [2]. More importantly, ELISA can only measure one analyte at a time. Also, other antigen-antibody interaction measurement techniques, such as AlphaScreen® (Perkin Elmer, Waltham, MA), have been recently developed [3–6]. However, there are limitations regarding the protein purity and size, as well as the buffer composition (terbium or europium chelates), which may affect the signal quality and add significant costs to the assay [7]. Protein microarrays can analyze thousands of analytes but at considerable cost, and the protein conformation, noise or measurement error may be difficult to calibrate [8]. Microsphere quantitative suspension array technology (qSAT), also known as Luminex or multiplex bead suspension array technology, represents an excellent alternative to ELISA and protein arrays, with high sensitivity and specificity [9]. qSAT is a flow cytometric assay that allows to test simultaneously up to 500 different analytes in a single reaction, reducing sample volume, labor, cost and enabling higher throughput [10]. qSAT bead-based assays use fluorescently encoded microspheres for capturing and detecting target molecules [9]. This platform is FDA-approved for diagnostics, has a high sensitivity, is versatile, and is amenable to screening large numbers of specimens [11,12].

A challenge of any immunoassay is its precision and robustness [13]. Notable sources of deviation may be run-to-run variability caused by differences in the test conditions and operator’s expertise. However, factors affecting assay performance have not been thoroughly and systematically evaluated. Classically, the assessment of the impact of variables in assay performance has been done in a series of one-factor-at-a-time experiments [14]. This sort of strategy is not capable of finding interactions between assay parameters that may affect both assay robustness and precision [15]. A more efficient approach to assay optimization is to utilize experimental designs to investigate the effects of several factors at once [16]. Fractional factorial designs of experiments are especially efficient during assay development because they can identify factors affecting assay performance and their interactions with a limited number of experiments [15].

In this study, we aimed to optimize assay parameters to maximize precision and robustness of an IgG qSAT assay developed *in-house* [17] against a multiplex panel of *P*. *falciparum* antigens. We analyzed variability considering the following assay factors, with two conditions each: coupling of the antigens to beads, sample predilution, temperature of incubation of samples with antigen-bead coupled, plate washing and operator expertise. We performed a fractional factorial design with the different assay conditions and measured IgG levels against seven antigens of different immunogenicities. We assessed the variability measured as median absolute deviation (MAD) for a combination of factors for each antigen and sample type. We also assessed the effect of combination of conditions on the MFI quantification range, and the potential interaction between conditions. We finally evaluated the minimum number of replicates for test samples, positive control and blanks to achieve good replicability.

## Materials and methods

### Study design

We assessed the effect of five qSAT assay conditions on assay variability. Assay conditions tested were selected based on our previous experience in the laboratory: beads coupled to antigens, performed once and stocked for the whole study (stock) vs. three different coupling sets performed during the study (several); sample predilution, frozen stock prepared at the beginning of the study (stock) vs. freshly prepared every assay day (daily); temperature of incubation of samples with antigen-bead, at 37°C vs. at 22°C; plate washing, automatic vs. manual; and operator expertise, expert vs. apprentice. Experiments were set up following a fractional factorial design including four factors (beads coupling, sample predilution, temperature of incubation and washing) with two conditions each. We performed a total of 64 assays (plates) (S1 Table). The operator expertise (expert vs. apprentice) was later included in the analysis. The experiments were conducted over three months.

### Samples and controls

We selected nine individual plasma samples of malaria-exposed donors from the ISGlobal repository based on data from previous field studies performed in the Manhiça District, Southern Mozambique [18]. Eight samples were from semi-immune adults with life-long exposure to malaria [19], and one sample from a child with clinical malaria [20]. We prepared a positive control with pooled plasma from 12 hyper-immune adults from Manhiça who participated in a clinical trial of intermittent preventive treatment conducted between 2003–2005 [21]. The immunological analysis of the samples was covered under human subject protocols approved by the National Mozambican Ethics Committee (117/CNBS/05, 85/CNBS/05 and 99/CNBS/05) and the Hospital Clínic of Barcelona Ethics Committee (CEIC 2008/4097). Written informed consent was obtained from all participants or their parents/guardians before collection of plasma samples used in research. All data were fully anonymized before we accessed them. To assess the MFI quantification range of the assay, we used an additional positive control consisting on a WHO reference standard prepared with pooled plasma from hyper-immune Kenyan adults [22]. Twenty-serial dilutions of the WHO reference standard against the study antigen panel were fitted in a non-linear regression model [23].

### Plate design

S1 Fig shows an example of plate design. Each assay plate included nine test samples assayed in 4 serial dilutions (1/100; 1/500; 1/20,000; 1/500,000), to ensure that at least one dilution would lay in the linear part of the antigen-specific titration curve. To assess the need of replicates, we duplicated samples in alternated dilutions (S1 Fig). We assayed the positive control in 8 serial dilutions (2.5 fold) starting at 1/50 and replicated it in alternated dilutions. Finally, 3 multiplex blanks (beads with the antigenic panel) were also included to measure the non-specific background reactivity.

### Recombinant proteins

We selected a panel of seven *P*. *falciparum* antigens with heterogeneous immunogenicities to ensure we accounted for variability in a wide range of antibody responses: the fragment 2 of region II of the 175 kDa erythrocyte binding protein (EBA-175 or PfF2) [24] and reticulocyte binding-like homologue protein 5 (Rh5) [25,26] expressed in *Escherichia coli* at ICGEB; the apical membrane antigen 1 (AMA-1) [27,28] and the merozoite surface protein 1 (MSP-1_42_), both from the FVO strain [27,29] and expressed in *E*. *coli* at WRAIR; the liver-stage antigen 1 (LSA-1) [30,31] and the sporozoite surface protein 2 (SSP2 or TRAP) [32,33] expressed in *Pichia Pastoris*, purchased from Protein Potential, LLC (Rockville, Maryland, USA); and a peptide with 48 aminoacids from the VAR2CSA inter domain 1 region, synthesized by GL Biochem (Shangai, China) [34]. Based on the readout of the positive control immunogenicities were considered high for AMA-1 and MSP-1_42_, medium for EBA-175 and Rh5 and low for LSA-1 and SSP2 [35,36].

### Microsphere covalent coupling

The assays were performed using the Luminex xMAP™ technology and a Luminex xMAP® 100/200 analyzer (Luminex Corp., Austin, Texas), which can analyze up to 80 MagPlex® microsperes. We calculated the amount of beads to be coupled to each antigen assuming the use of 1,000 beads/well/antigen for test samples, positive control and blanks. We washed MagPlex® 6.5μm COOH-microspheres twice with 250μL of distilled water using a magnetic separator, and re-suspended them to a final concentration of 10,000 beads/μL by short vortexing and sonication for 20 sec. Microspheres were re-suspended in 80μL bead activation buffer (100 mM Monobasic Sodium Phosphate, pH 6.2, Sigma, Tres Cantos, Spain) by vortexing and sonication for 20 sec, and activated using 10μL of 50 mg/mL Sulfo-NHS (N-hydroxysulfosuccinimide) and 10μL of 50 mg/mL EDC (1-Ethyl-3-[3-dimethylaminopropyl] carbodiimide hydrochloride) (Thermo-fisher Scientific Inc., MA, USA) simultaneously added to the reaction tubes. We mixed reaction tubes by vortex and incubated for 20 min, at room temperature (RT), in a rotary shaker and protected from light. We washed microspheres twice with 250μL 50 mM MES (morpholineethane sulfonic acid) (Sigma, Tres Cantos, Spain) pH 5.0 for AMA-1, MSP-1_42_, Rh5 and VAR2CSA, or phosphate buffered saline (PBS) pH 7.4 for EBA-175, LSA-1 and SSP2, and resuspended to a 10,000 beads/μL concentration by vortexing and sonication for 20 sec. A prior selection of the optimal buffer and protein concentration for the coupling of each antigen was performed testing both buffers and serial concentrations of the proteins (10, 30, 50, 100 μg/mL) and assaying the coupled beads against a hyperimmune plasma. Conditions giving coupling saturation were selected (data not shown). Finally, to coat the beads with antigens, we added the appropriate concentration of the corresponding protein (10 μg/mL SSP2; 20 μg/mL AMA-1, MSP-1_42_, EBA-175 and LSA-1; 30 μg/mL Rh5; and 1,760 μg/mL VAR2CSA) to each reaction tube in 500μL MES pH 5.0 or PBS pH 7.4 (10,000 beads/μL), depending on the antigen. Reaction tubes were left at 4°C on a rotatory shaker overnight and protected from light. Next day, microspheres were blocked with 250μL PBS-BN (PBS with 1% Bovine serum albumin [BSA] [Santa Cruz] and 0.05% sodium azide [Sigma, Tres Cantos, Spain]) in agitation during 30 min at RT and protected from light. We centrifuged and washed the beads twice with 250μL of PBS-BN and resuspended in 500μL of PBS-BN to be quantified on a Guava PCA desktop cytometer (Guava, Hayward, CA, USA). To create our multiplex antigen panel, we combined equal amounts of each coupled-bead-protein in tubes at 2,500 beads/μL, and aliquoted and stored them at 4°C protected from light. Antigen-coupled beads were stored during the study period, being stable for at least three months.

### qSAT assay procedure

All samples were prediluted at double concentration of the final assay dilution with PBS-BN. Stock prediluted samples were stored at -80°C for a maximum of 3 months and freshly prediluted samples were prepared each day by thawing the corresponding sample and diluting it with PBS-BN. We added antigen-coupled beads to a 96-well μClear® flat bottom plate (Greiner Bio-One) in multiplex (1,000 microspheres/analyte/well) and resuspended them in a volume of 50 μL of PBS-BN. Next, we added 50 μL of test samples and positive control serial dilutions to multiplex wells. We filled blank wells with PBS-BN. Plates were incubated for 1 h at 22°C or 37°C (depending on the study group the plate had been assigned to) in a rotatory shaker at 600 rpm and protected from light. After incubation, we washed plates three times with 200μL/well of wash buffer (PBS-Tween 20 0.05%, Sigma, Tres Cantos, Spain) with 1 min lapse in between, using a magnetic washer, manual (Millipore, ref. 40–285) or automatic (Bio-Plex Pro II Wash Station, BioRad) set up with the same procedure as manual washing, depending on the group the plate had been assigned to. Afterwards, we added 100μL of biotinylated anti-human IgG (Sigma, Tres Cantos, Spain) diluted 1:2,500 in PBS-BN to all wells, and incubated 45 min, at RT, in agitation at 600 rpm and protected from light. The plate was washed three times as before, and 100μL of streptavidin-R-phycoerythrin (Sigma, Tres Cantos, Spain) diluted 1:1,000 in PBS-BN were added to all wells and incubated 25 min, at RT, 600 rpm and protected from light. We washed plates three times and wells were resuspended in 100 μL/well of PBS-BN. Plates were then covered protected from light and stored at 4°C overnight to be read the next day using the Luminex xMAP® 100/200 analyzer, and at least 50 microspheres per analyte were acquired per sample.

Antigen-specific serial dilution curves were prepared in a separate experiment using the WHO reference standard [22], and were fitted using a non-linear 4-parameter log-logistic (4-PL) function with data points log_10_ transformed via [23]:

$$f(x;b,c,d,e)=c+\frac{d-c}{1+10^{b(x-e)}},$$

where “b” is the slope at the inflection point, “c” is the lower asymptote, “d” is the upper asymptote and “e” is the concentration at the inflection point.

Finally, data files including crude median fluorescent intensity (MFI) and bead counts for each analyte and well were exported.

### Statistical analysis

The distributions of MFIs of test samples, positive control dilutions and blanks were compared through t-test. We conducted exploratory graphical analysis using spaghetti plots of the log_10_MFI against dilution series for the antigen-specific positive control curves. These plots were stratified by each one of the 2x2 conditions combinations.

The assessment of the MFI quantification range was performed using the curves generated with 20 serial dilutions of the WHO reference standard, and was based on the coefficient of variation method for estimating the limits of quantification using a cutoff of 20% [37,38].

To assess the variability associated with each factor and combination of factors, we calculated the MAD for each dilution point and antigen, for test samples, positive control and blanks. To assess factors that could impact assay variability, we fitted linear regression models for each combination of conditions to measure variability as log_10_MAD of log_10_MFI or MFI quantification range with each of the factors of interest as predictor, separately and jointly, and interaction terms were also assessed. Replicates for each type of sample and antigen were assessed using the Intraclass Correlation Coefficient (ICC) [39] and Bland-Altman graphs [40]. P-values were considered significant if < 0.05 and p-values between 0.05 and 0.10 “marginally” significant. When appropriate, p-values were corrected for multiple testing (designated here as p-adjusted [p-adj]) by Benjamini & Hochberg method [41]. All the analyses were performed in R software version 3.2.2.

## Results

### Impact of assay conditions on variability depends on antigen immunogenicity and sample concentration

**Fig 1:**
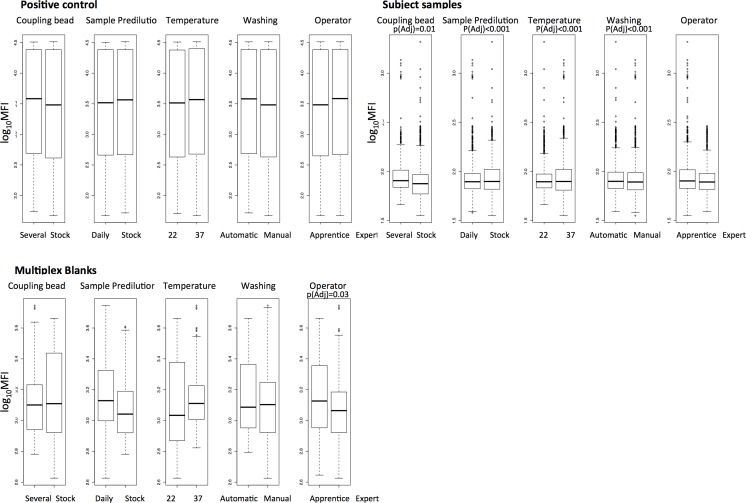
Boxplots of log_10_MFI distribution comparing pairwise assay conditions. Conditions compared were: antigen-bead coupling, prepared once for the whole study (stock) vs. three times along the study (several); sample predilution prepared once for the whole study (stock) vs. daily performed (daily); temperature of sample-beads incubation (22°C vs. 37°C); plate washing (Automatic vs. Manual) and operator expertise (Expert vs. Apprentice). All antigens and sample serial dilutions were included in the analysis. P-values were estimated through t-test and adjusted for multiple testing by Benjamini & Hochberg. Only significant p-values are shown.

**Fig 2:**
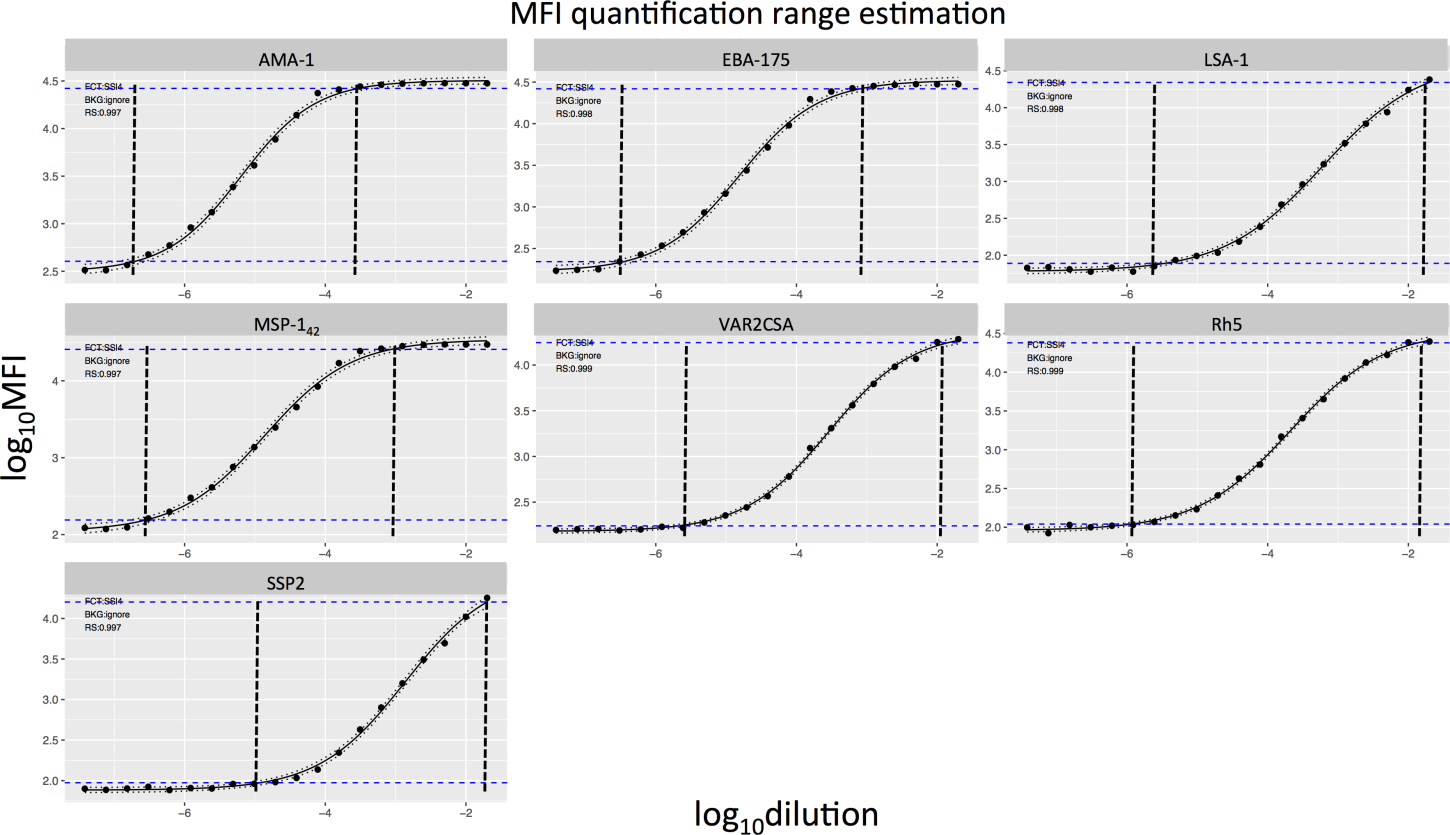
WHO reference standard curves against each of the antigens in the multiplex panel. Horizontal blue dashed lines represent the MFI quantification range (where the coefficient of variation is below 20%). The vertical black dashed lines encompass more reliable values of the assay output.

Differences in the distribution of overall MFIs between assay conditions and operator experience, taking all antigen data and serial dilutions together, were analyzed for positive control, test samples, and blanks (Fig 1). The MFI distribution of test samples changed significantly depending on the antigen-bead coupling (p-adj = 0.01), sample predilution (p-adj < 0.001), temperature of incubation of samples with antigen-bead (p-adj < 0.001) and plate washing (p-adj < 0.001). The distribution of blank MFIs varied depending on the operator expertise, having higher background signal when the operator was an apprentice (p-adj = 0.035). No differences between assay conditions were found for the positive control. The assay MFI quantification range was different depending on the antigen, as shown by the antigen-specific curves prepared in another set of experiments with serial dilutions of the WHO reference standard (Fig 2). Therefore, we analyzed the data taking into consideration each antigen MFI quantification range.

**Fig 3:**
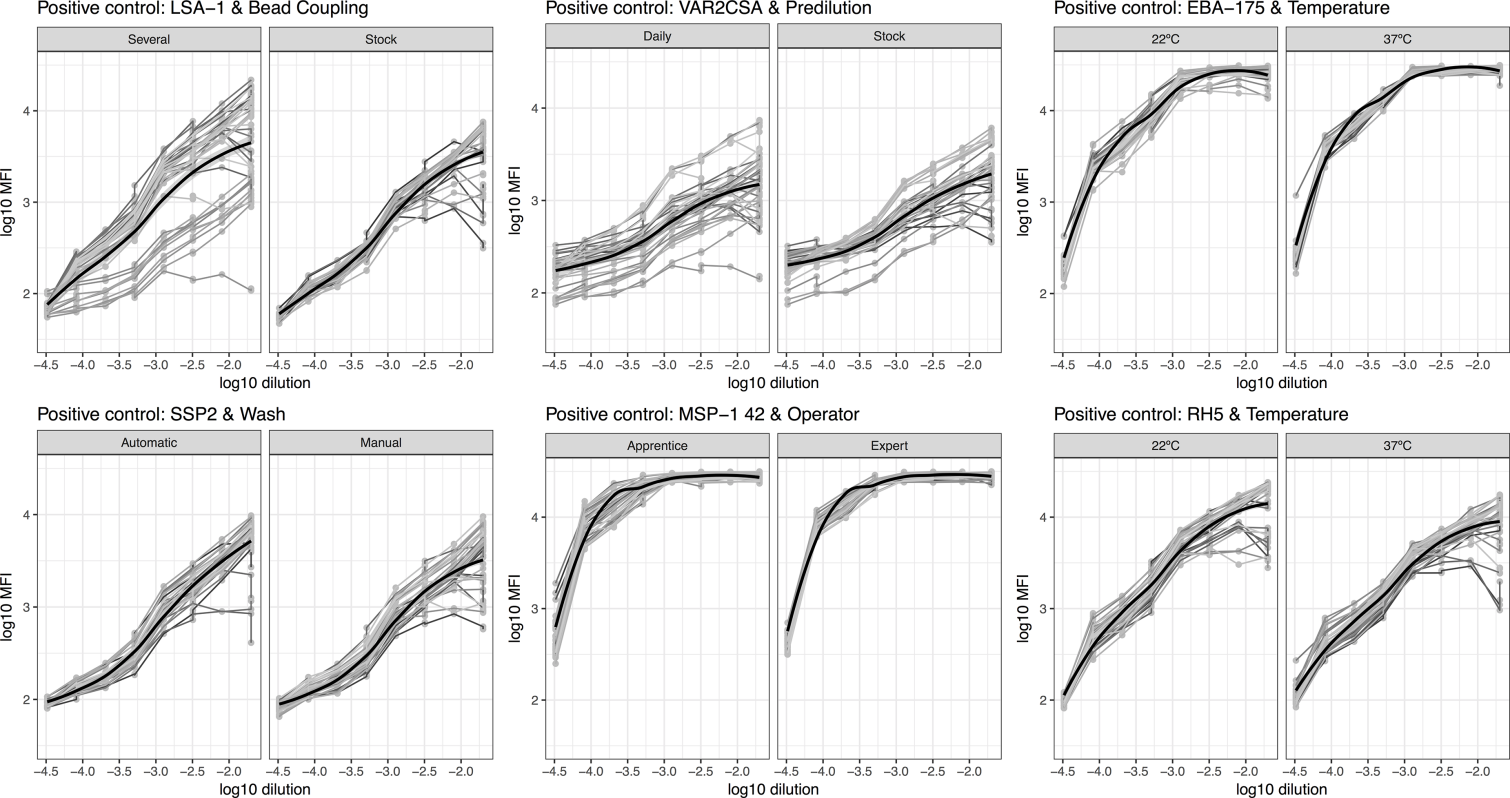
Antigen-specific log_10_MFI levels of positive control serial dilutions by assay conditions. Spaghetti plots represent examples of positive control serial dilution MFIs against different antigens and in different assay conditions: Antigen-bead coupling (stock vs. several) for LSA-1; Sample predilution (stock vs. daily) for VAR2CSA; Temperature of sample-beads incubation (22°C vs. 37°C) for EBA-175 and Rh5; Plate washing (automatic vs. manual) for SSP2 and operator expertise (apprentice vs. expert) for MSP-1_42_. Grey lines correspond to data from each plate.

Positive control serial dilution curves showed different MFI variability across dilutions and antigens. A single coupling of antigens to the beads (stock), incubation at 37°C, automatic washing and assay performed by an expert operator resulted in reduced MFI dispersion at more concentrated sample dilution in low immunogenic antigens, such as VAR2CSA and LSA-1, and at less concentrated sample dilution in high immunogenic antigens, such as MSP-1_42_ or AMA-1 (Fig 3 and S2 Fig).

### Evaluation of assay variability associated to combined assay conditions

**Fig 4:**
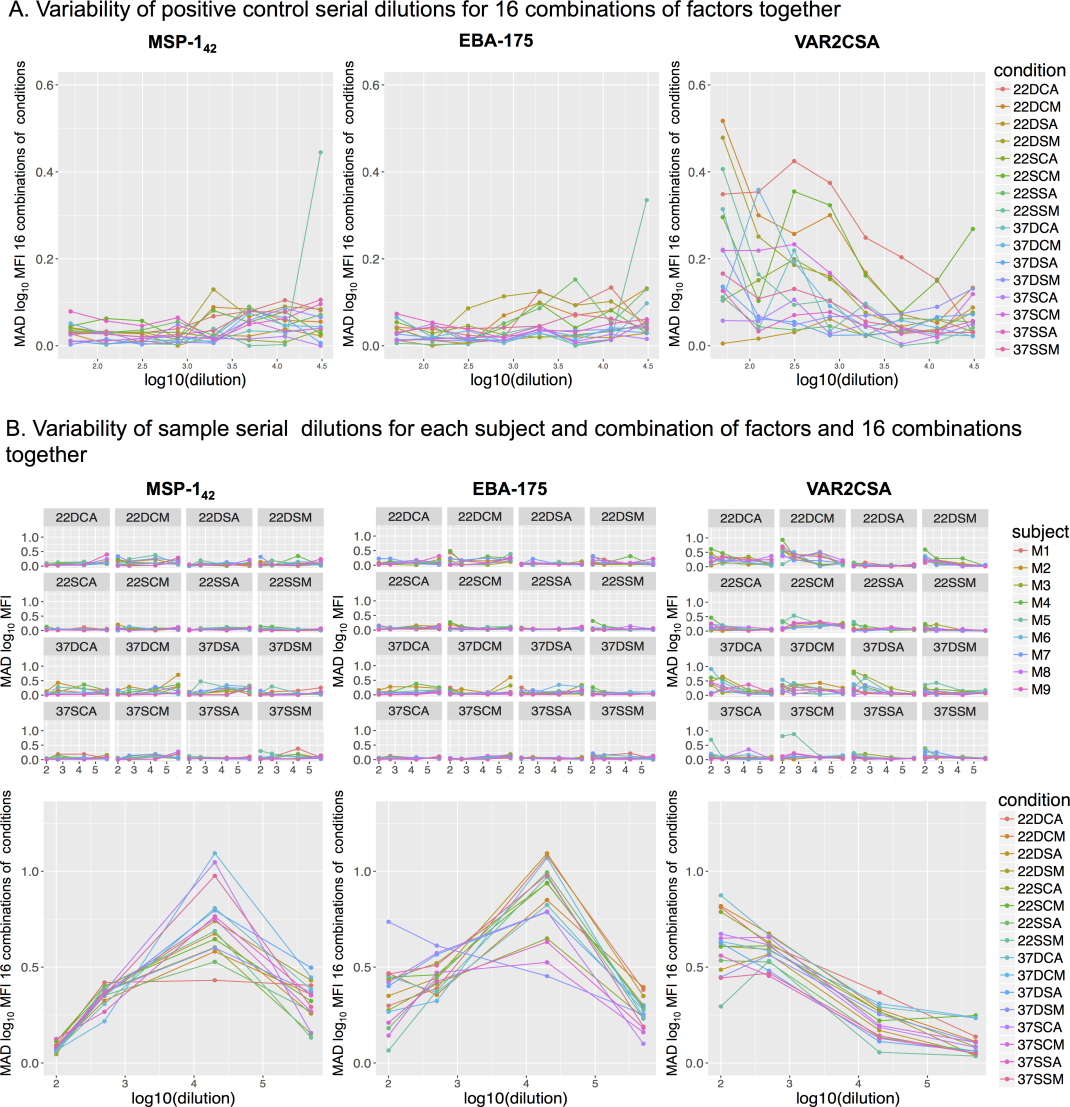
MAD of log_10_MFI against MSP-1_42_, EBA-175 and VAR2CSA, in the 16 combinations of assay conditions. A) Positive control in 8 serial dilutions (2.5-fold starting at 1/50), where each line in the graphs represents one of the 16 combinations of the 4 assay conditions; B) Top: Test samples in 4 dilutions (1/100, 1/500, 1/20,000, 1/500,000) for each of the 16 combinations of conditions, where each line in the graphs represents one of the 9 subjects analyzed; bottom: 16 combinations of the 4 assay conditions for all test samples analyzed together, where each line in the graphs represents one of the combinations. The 16 combinations of the 4 assay conditions are designated by the numbers and letters corresponding to: temperature of incubation (37 = 37°C and 22 = 22°C), sample predilution (D = daily and S = stock), beads coupling (S = stock and C = three times along the study), and plates washing (A = automatic and M = manual).

To quantify and compare the variability associated with each assay condition, we calculated the MAD of log_10_MFI for the positive control serial dilutions and test samples against each antigen and at each dilution point. Variability associated to single assay conditions varied across antigens and dilutions (S3 Fig). In addition, results on single assay conditions could be affected by other conditions, thus this analysis did not allow us to conclude which conditions reduced variability. On the other hand, the fractional factorial design allowed us to assess the optimal combination of conditions with the minimum assay variability. By merging all combinations of conditions (coupling, predilution, temperature and washing) we obtained 16 possible combinations (Fig 4). Highly immunogenic antigens such as MSP-1_42_ showed higher variability, measured as MAD levels, at more diluted concentrations of the positive control (Fig 4A) or test samples (Fig 4B). Inversely, low immunogenic antigens such as VAR2CSA had more variability at higher concentrations of the positive control or test samples (Fig 4). Overall, the highest assay variability for each antigen lied on the linear part of its antigen-specific positive control titration curve, which was marked by the curve MFI quantification range (Fig 2). Therefore, antigens with different immunogenicities had dilution-dependent variability at the analyzed conditions.

**Fig 5:**
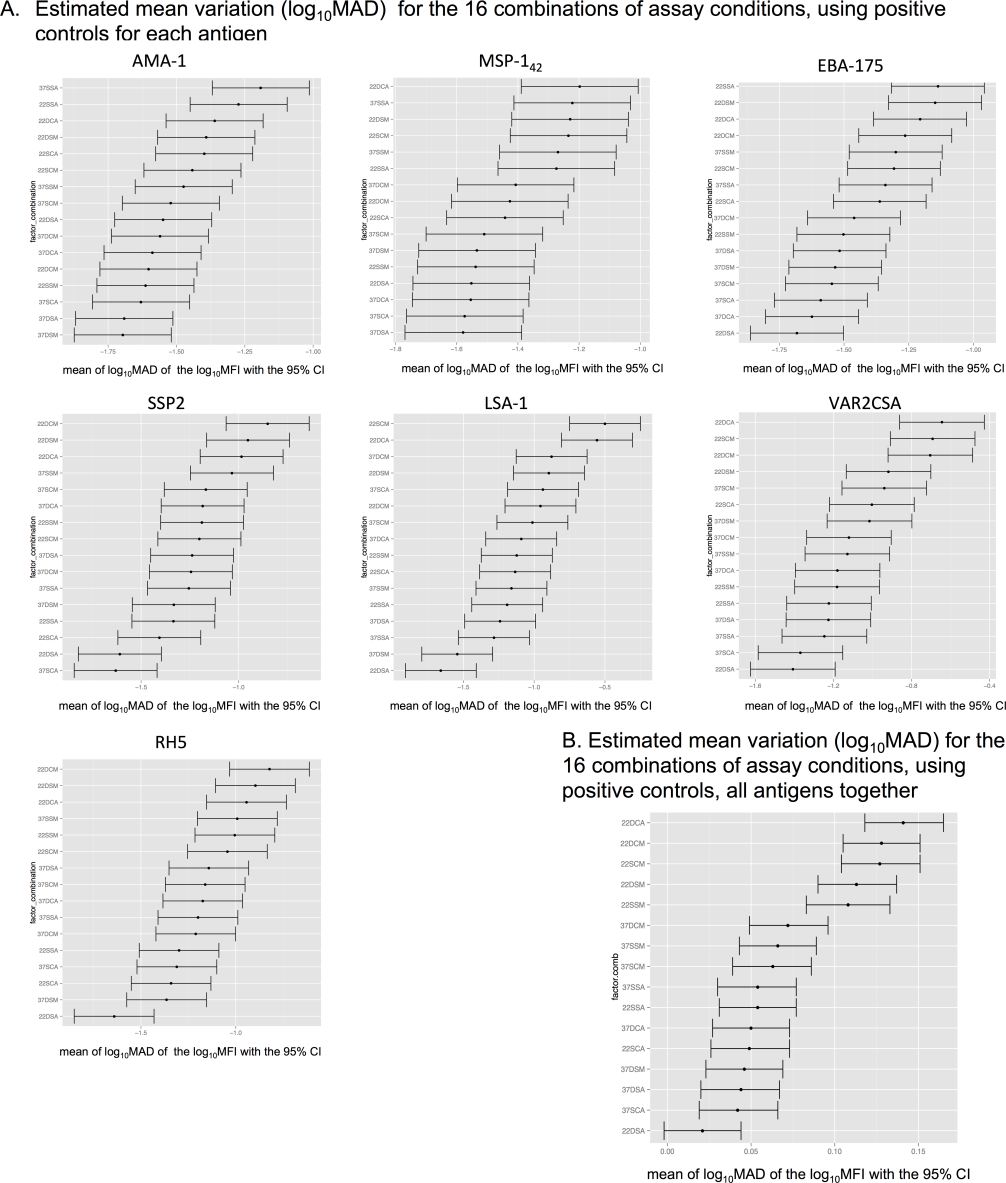
Ranked log_10_MAD of log_10_MFI for the 16 combinations of the four assay conditions in the positive control. The x-axis shows the mean log_10_MAD of log_10_MFI and 95% confidence intervals. A) By antigen and B) Combining all antigens. The y-axis shows combinations of the following conditions, ordered by the value of the mean log_10_MAD: temperature of sample-bead incubation (37 = 37°C and 22 = 22°C), sample predilution (D = daily and S = stock), beads coupling (S = single and C = three or more combined), and plate washing (A = automatic and M = manual).

To evaluate the impact of each combination of conditions per antigen, we estimated the mean MAD of all positive control serial dilutions and ranked them (Fig 5A). Five out of the 7 antigens showed that the combination of assay conditions that resulted in less variability was stock coupling with daily sample predilution and automatic washing (DSA) (Fig 5A). However, the combination resulting in largest assay variability varied by antigen. Nevertheless, when performing the analysis for all antigens together, doing several couplings, daily samples predilution, sample-beads incubation at 22°C and automatic washing (22DCA), showed the highest variability (Fig 5B); but, a unique coupling, daily samples predilution, samples-beads incubation at 22°C and automatic washing (22DSA) showed the lowest variability. All together, the combinations of assay conditions giving the highest and lowest variability differed only by the bead coupling (unique vs. several), suggesting that this factor is an important source of variability.

### Impact of assay conditions and paired combinations on assay variability

We next assessed the impact of assay conditions and their combination in pairs on assay variability, measured as log_10_MAD of log_10_MFI, by linear regression models with single or combined conditions as predictors. Separate regression models were built for the positive control, test samples and blanks. The results in Tables 1 and 2 illustrate the effect of single assay conditions and pair combinations of assay conditions on assay variability, measured as log_10_MAD of log_10_MFI. Tables show the regression coefficients, p-values and interaction p-values. For instance, in Table 1 we are analyzing the effect of bead coupling on assay variability when using the positive control. In this model we are including 112 measurements that come from the 2 conditions of coupling [bead (stock) vs. three times along the study (several)] x 8 serial dilutions of the positive control x 7 antigens in the panel. Then, we have analyzed the effect of sample predilution on bead coupling and its combined effect on assay variability. In this model we are including 224 measurements that come from the 2 conditions of coupling [bead (stock) vs. three times along the study (several)] x 8 serial dilutions x 7 antigens x 2 condition of sample predilution [once (stock) vs. daily (daily)].

**Table 1:** Linear regression models assessing the impact of combination of assay conditions on assay variability (log_10_MAD of log_10_MFI). Regression coefficients and p-values for one and two factors, all dilutions and antigens included. Bead coupling performed once at the beginning of the study (stock) or three times along the study (several); Sample predilution prepared at once (stock) or daily (daily); Sample-bead incubation temperature at 22°C or 37°C; Plate washing in an automated washer (automatic) or manually (manual); and Operator expertise, apprentice or expert. A) Positive control, 2 conditions, 8 dilutions and 7 antigens (N = 112); B) Subject samples, 2 conditions, 4 dilutions and 7 antigens (N = 56) and C) Multiplex blanks, 2 conditions and 7 antigen (N = 14). P-values adjusted for multiple testing by Benjamini & Hochberg (p-Adj). Significant p-values are shown in bold.

**A. Positive control**															
	**Bead Coupling**			**Sample Predilution**			**Incubation Temp.**			**Washing**			**Operator**		
**Assay conditions**	**Stock**	**p-value**	**N**	**Stock**	**p-value**	**N**	**37°C**	**p-value**	**N**	**Manual**	**p-value**	**N**	**Apprentice**	**p-value**	**N**
		**(p-Adj)**			**(p-Adj)**			**(p-Adj)**			**(p-Adj)**			**(p-Adj)**	
Stock Bead coupling	-0.055	0.31	112	-0.02	0.62	224	-0.043	0.28	224	0.1	0.01	224	0.053	0.19	224
		(0.44)			(0.73)			(0.43)			(0.09)			(0.34)	
Stock Sample predilution	-0.051	0.22	224	-0.034	0.56	112	-0.066	0.15	224	0.081	0.058	224	0.042	0.32	224
		(0.34)			(0.68)			(0.33)			(0.27)			(0.45)	
37°C Incubation	-0.14	**< 0.001**	224	-0.077	0.09	224	-0.087	0.17	112	0.015	0.74	224	0.095	0.04	224
		**(<0.001)**			(0.25)			(0.33)			(0.81)			(0.21)	
Manual wash	-0.051	0.2	224	-0.0034	0.94	224	-0.09	0.056	224	0.11	0.052	112	-0.009	0.83	224
		(0.34)			(0.94)			(0.21)			(0.22)			(0.86)	
Apprentice Operator	-0.07	0.08	224	-0.037	0.37	224	-0.072	0.12	224	0.08	0.054	224	0.028	0.64	112
		(0.25)			(0.48)			(0.31)			(0.22)			(0.73)	
**B. Test samples**															
	**Bead Coupling**			**Sample Predilution**			**Incubation Temp.**			**Washing**			**Operator**		
**Assay conditions**	**Stock**	**p-value**	**N**	**Stock**	**p-value**	**N**	**37°C**	**p-value**	**N**	**Manual**	**p-value**	**N**	**Apprentice**	**p-value**	**N**
		**(p-Adj)**			**(p-Adj)**			**(p-Adj)**			**(p-Adj)**			**(p-Adj)**	
Stock Bead coupling	-0.032	0.74	56	-0.056	0.42	112	-0.0077	0.91	112	0.033	0.63	112	0.06	0.63	112
		(0.91)			(0.91)			(0.91)			(0.91)			(0.91)	
Stock Sample predilution	-0.021	0.76	112	-0.041	0.66	56	0.011	0.88	112	0.025	0.7	112	0.05	0.7	112
		(0.91)			(0.91)			(0.91)			(0.91)			(0.91)	
37°C Incubation	-0.064	0.38 (0.91)	112	-0.069	0.32	112	0.015	0.88	56	0.009	0.9	112	0.085	0.9	112
		(0.91)			(0.91)			(0.91)			(0.91)			(0.91)	
Manual wash	-0.029	0.67	112	-0.044	0.51	112	0.011	0.88	112	0.032	0.73	56	0.054	0.73	112
		(0.91)			(0.91)			(0.91)			(0.91)			(0.91)	
Apprentice Operator	-0.036	0.6	112	-0.035	0.61	112	0.023	0.75	112	0.033	0.62	112	0.058	0.62	56
		(0.91)			(0.91)			(0.91)			(0.91)			(0.91)	
**C. Blanks**															
	**Bead Coupling**			**Sample Predilution**			**Incubation Temp.**			**Washing**			**Operator**		
**Assay conditions**	**Stock**	**p-value**	**N**	**Stock**	**p-value**	**N**	**37°C**	**p-value**	**N**	**Manual**	**p-value**	**N**	**Apprentice**	**p-value**	**N**
		**(p-Adj)**			**(p-Adj)**			**(p-Adj)**			**(p-Adj)**			**(p-Adj)**	
Stock Bead coupling	-0.18	0.11	14	0.0082	0.91	28	0.048	0.55	28	0.1	0.1	28	0.071	0.32	28
		(0.33)			(0.91)			(0.81)			(0.33)			(0.59)	
Stock Sample predilution	-0.16	**0.03**	28	0.035	0.69	14	0.085	0.3	28	0.064	0.33	28	0.02	0.81	28
		(0.15)			(0.84)			(0.59)			(0.59)			(0.84)	
37°C Incubation	-0.24	**0.006**	28	(0.023	0.78	28	0.13	0.27	14	0.085	0.25	28	0.13	0.12	28
		(0.1)			(0.84)			(0.59)			(0.59)			(0.33)	
Manual wash	-0.14	**0.03**	28	0.058	0.38	28	0.16	**0.04**	28	0.025	0.78	14	0.023	0.72	28
		(0.15)			(0.63)			(0.17)			(0.84)			(0.84)	
Apprentice Operator	-0.2	**0.008**	28	0.02	0.81	28	0.18	**0.03**	28	0.048	0.46	28	0.044	0.67	14
		(0.1)			(0.84)			(0.15)			(0.72)			(0.84)	

**Table 2:** Linear regression models assessing the impact of combination of assay conditions on the MFI quantification range. Regression coefficients and p-values for one and two factors, all dilutions and antigens included. Bead coupling performed once at the beginning (stock) or three times along the study (several); Sample predilution, prepared at once (stock) or daily (daily); Sample-bead incubation temperature at 22°C or 37°C; Plate washing in an automated washer (automatic) or manually (manual); and Operator expertise, Apprentice or Expert. Regression models for positive control, 2 conditions, 8 dilutions and 7 antigens (N = 112). P-values adjusted for multiple testing by Benjamini & Hochberg (p-Adj). Significant p-values are shown in bold.

	Bead Coupling			Sample Predilution			Incubation Temp.			Washing			Operator		
Assay conditions	Stock	p-value	N	Stock	p-value	N	37°C	p-value	N	Manual	p-value	N	Apprentice	p-value	N
		(p-Adj)			(p-Adj)			(p-Adj)			(p-Adj)			(p-Adj)	
Stock Bead coupling	-0.07	0.15	112	0.02	0.75	448	-0.06	0.18	448	-0.07	0.12	448	-0.12	**0.01**	448
		(0.22)			(0.84)			(0.24)			(0.22)			(0.05)	
Stock Sample predilution	-0.07	0.15	448	0.01	0.81	112	-0.07	0.15	448	-0.07	0.15	448	-0.13	**<0.01**	448
		(0.22)			(0.84)			(0.22)			(0.22)			(0.05)	
Incubation 37°C	-0.07	0.18	448	0.01	0.81	448	-0.07	0.15	112	-0.07	0.15	448	-0.15	**<0.01**	448
		(0.24)			(0.84)			(0.22)			(0.22)			(0.05)	
Manual Wash	-0.07	0.12	448	0.01	0.81	448	-0.07	0.15	448	-0.07	0.15	112	-0.13	**<0.01**	448
		(0.22)			(0.84)			(0.22)			(0.22)			**(0.05)**	
Operator apprentice	-0.05	0.34	448	0	0.92	448	-0.1	**0.047**	448	-0.08	0.1	448	-0.13	**<0.01**	112
		(0.42)			(0.92)			(0.09)			(0.22)			(0.05)	

For the positive control, we found that the combination of bead couplings performed once at the beginning of the study (stock) and incubation of samples with antigen-bead at 37°C, reduced assay variability (p<0.001) (Table 1). For the test samples we did not find any effect of single or combined conditions on assay variability (Table 1). For the blanks, we observed a weak reduction on variability if stock coupling was combined with stock predilution (p = 0.03), or blank-bead incubation at 37°C (p = 0.006), or manual washing (p = 0.03) or apprentice operator (p = 0.008), or if incubating at 37°C was combined with an apprentice operator (P = 0.03), or manual wash (p = 0.04). However the effect on variability of all these combinations was lost after adjusting for multiple comparisons (Table 1).

**Fig 6:**
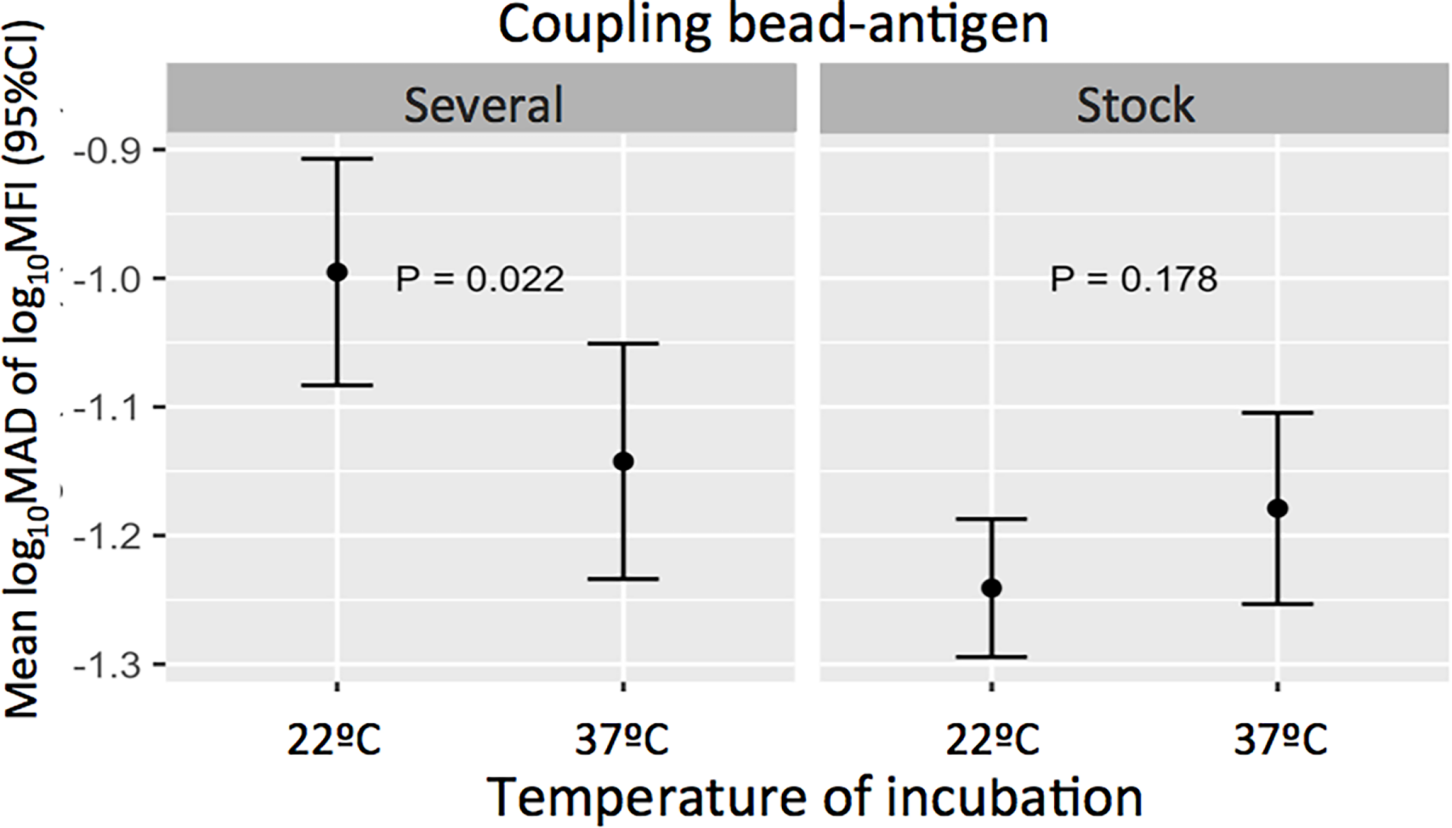
Impact of temperatures of incubation across coupling conditions on the positive control assay variability. Variability was measured as mean log_10_MAD of log_10_MFI with 95% confidence intervals. P-values correspond to the difference between variability associated to each temperature of incubation when beads were coupled once (stock) or three times along the study (several).

We also analyzed whether a condition could modify the variability associated with another condition. When working with the positive control, the effect of temperature of incubation on assay variability was statistically significantly different across coupling categories (p-_Interaction_ = 0.008). Specifically, when the coupling was performed at different times during the study and the incubation was performed at 22°C, the variability (log_10_MAD of log_10_MFI) was higher compared to incubating at 37°C (Fig 6). Nevertheless, when using a unique coupling, the variability was not different between temperatures of incubation (Fig 6).

### Impact of assay conditions and paired combinations on the MFI quantification range

We tested the impact of assay conditions and their combination in pairs on the assay MFI quantification range by linear regression models with single or combined conditions and their interactions as predictors. This analysis was done for the positive control. We found that beads coupling, sample predilution, temperature of incubation, and washing, did not affect the MFI range (Table 2). However, if an apprentice operator performed the assay, the MFI range was significantly reduced (p = 0.05), and this reduction was maintained no matter the other assay conditions. The effect of washing on the MFI quantification range was statistically significantly different across operator categories (p-_Interaction_ = 0.04). Specifically, when the washing was manual and an apprentice performed the assay, the MFI range was reduced compared to assay performed by an expert operator.

### Assessment of the minimum number of replicates required

**Fig 7:**
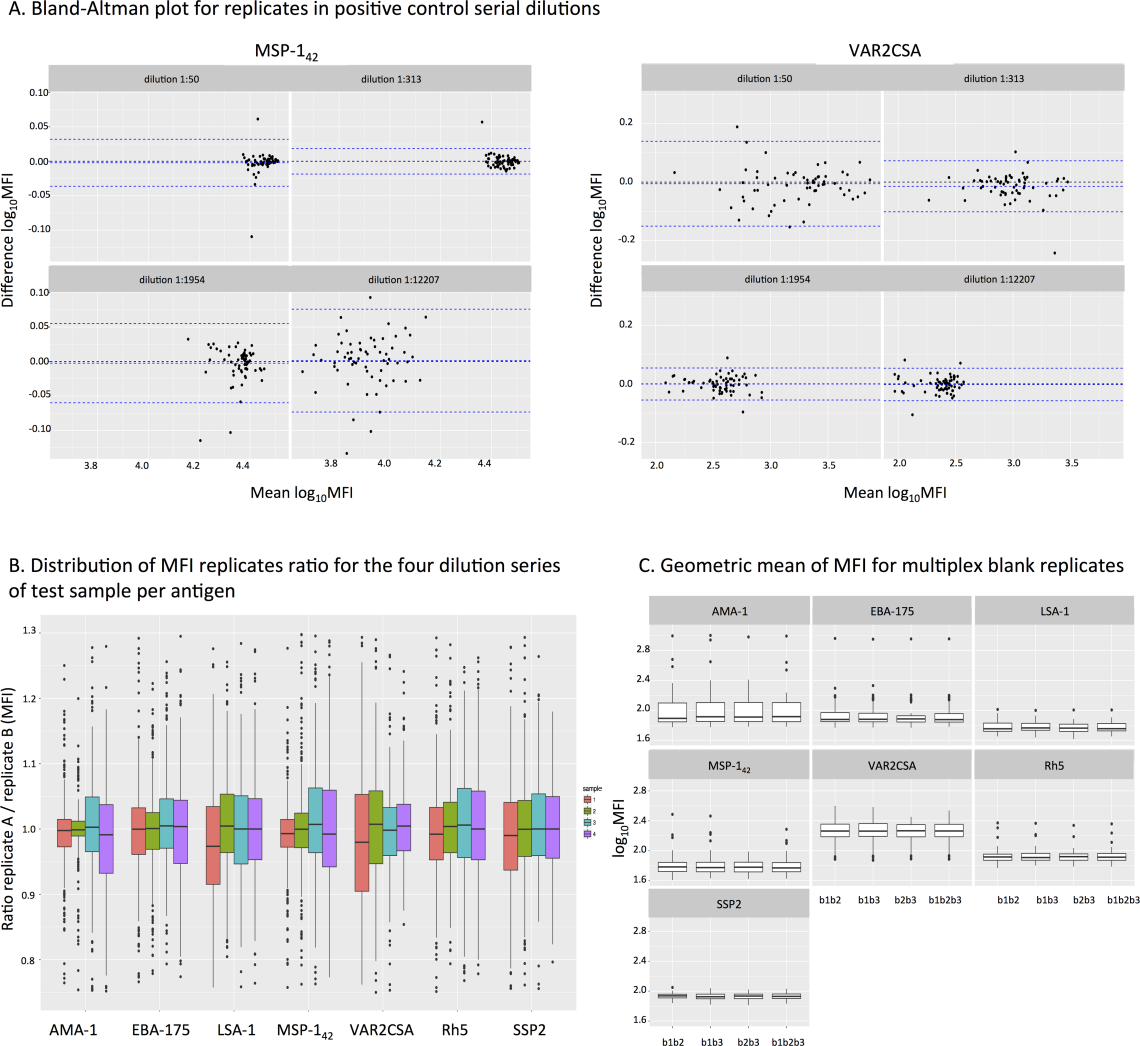
Assessment of replicates performance in the positive control, test samples and blanks. A) Bland-Altman plots showing the differences of positive control serial dilution replicates against its mean for MSP-1_42_ and VAR2CSA. Dashed blue lines show the 95% confidence interval of the differences. B) Boxplots representing the distribution of replicate MFI ratios for the four dilutions of test samples and per antigen. C) Boxplots representing the distribution of pairs and triplets of blanks per antigen (b1 blank1, b2 blank 2, and b3 blank3). Boxplots represent the mean and interquartile range.

To optimize the plate design, we assessed the advantage of using three instead of two blank controls. We also assessed whether testing duplicates of samples and positive control serial dilutions added information to the assay. Positive controls were assayed in 8 serial dilutions and samples in 4 serial dilutions with replicates in alternated positions (S1 Fig). The ICCs between replicates of a given dilution of the positive control ranged from 0.89 to 0.99 (Table 3), which is considered very good reliability [39]. To further investigate the need of replicates in the positive control serial dilutions, we performed Bland-Altman plots for each antigen (Fig 7A and S4 Fig). Considering all antigens together, 95% confidence intervals of the differences were between 0.2 and -0.2. We performed a replicate MFI ratio analysis for test samples. Results showed a minimal difference but of varying distribution along sample dilutions depending on antigen immunogenicity (Fig 7B), being higher on the more diluted samples for high immunogenic antigens (AMA-1 and MSP-1_42_) and lower on the more concentrated samples for low immunogenic antigens (VAR2CSA). This minimal difference between replicates of test samples agrees with the results from the positive control ICC analysis. Taking together, these results suggest that duplicates in positive control and samples replicate very well, therefore investing in more dilutions instead of replicates may improve the assay precision by increasing the likelihood of MFIs falling in the linear part of the antigen-specific curve.

**Table 3:** Interclass correlation coefficient (ICC) between replicates. A) ICC for the replicates of the positive control serial dilutions (2.5-fold starting at 1:50 with replicates in alternate positions); and B) ICC for the three blanks per plate. ICCs are assessed for each antigen and all dilutions combined.

A. Positive control replicates			B. Blank replicates				
Antigen	Duplicated dilution	ICC	Antigen	ICC (all)	ICC (1vs.2)	ICC (1vs.3)	ICC (2vs.3)
AMA-1	1:50	0.94	AMA-1	0.67	0.65	0.71	0.64
AMA-1	1:313	0.98	EBA-175	0.86	0.81	0.82	0.97
AMA-1	1:1954	0.98	LSA-1	0.81	0.83	0.73	0.87
AMA-1	1:12207	0.97	MSP-1_42_	0.72	0.69	0.66	0.91
EBA-175	1:50	0.94	VAR2CSA	0.97	0.96	0.96	0.99
EBA-175	1:313	0.96	Rh5	0.76	0.77	0.71	0.82
EBA-175	1:1954	0.94	SSP2	0.69	0.72	0.73	0.63
EBA-175	1:12207	0.97					
LSA-1	1:50	0.99					
LSA-1	1:313	0.99					
LSA-1	1:1954	0.99					
LSA-1	1:12207	0.98					
MSP-1_42_	1:50	0.90					
MSP-1_42_	1:313	0.97					
MSP-1_42_	1:1954	0.89					
MSP-1_42_	1:12207	0.94					
VAR2CSA	1:50	0.98					
VAR2CSA	1:313	0.98					
VAR2CSA	1:1954	0.99					
VAR2CSA	1:12207	0.98					
Rh5	1:50	0.97					
Rh5	1:313	0.95					
Rh5	1:1954	0.90					
Rh5	1:12207	0.96					
SSP2	1:50	0.97					
SSP2	1:313	0.95					
SSP2	1:1954	0.92					
SSP2	1:12207	0.90					

Additionally, to establish the minimum optimal number of blank replicates, we calculated the ICCs for all blanks together and each combination of blanks (Table 3). ICCs between blank replicates were lower than for positive control replicates, ranging from 0.69–0.97 and independently of antigen immunogenicity. However, the ICC of three blanks was similar to the ICC of two blanks, and the distribution of the mean and interquartile ranges of the different combinations of blank replicates showed no difference between using two or three replicates (Fig 7C).

## Discussion

One of the major challenges of large immunological studies is to have precise and robust high-throughput techniques to generate reliable results [13]. Herein we systematically assessed potential sources of variability in operator’s performance and conditions of an IgG qSAT assay against seven *P*. *falciparum* antigens with diverse immunogenicities aiming to establish the most optimal parameters. To our knowledge, this is the first time that a fractional factorial design has been used to assess the effect of several assay conditions on outcomes [16]. Fractional factorial design reduces the total number of experiments while determining the parameters that give the maximum sensitivity range with the maximum accuracy, providing valid results for multifactorial effects [42].

The qSAT multiplexed format has the advantage of allows analyzing antibody responses to up to 500 antigens in one single reaction [10], although the number of antigens that can be analyzed at a time depends on the type of microspheres, instrument and technology used. By testing MFI mean differences with pooled antigens and dilutions, we showed that among test samples there were different effects on assay variability depending on the coupling (stock vs. several), sample predilution (stock vs. daily), temperature of incubation of samples with antigen-bead (22°C vs. 37°C) and plate washing (automatic vs. manual). Also, variability of blanks was different depending on the operator expertise. However, in this crude analysis, antigen immunogenicities were not taken into account. In fact, we showed that the MFI quantification range for each antigen was different (Fig 2). As a result, the same sample or control would have different optimal working dilutions for a given antigen.

In the qSAT assays, it is common practice to extrapolate fluorescence data to concentration [43–45], but analytes at low concentration might be out of standard curve detection limits, making difficult to do the estimation. Fluorescence-based analysis have higher statistical power than concentration-based analysis, thus are a better choice for assigning statistical significance to main effects. Also, fluorescence responses are measured independently from a standard curve, reflecting actual variation, while estimated concentration values are dictated by the precision of the standard curve [44]. For all these reasons, we assessed the effect of multiple assay conditions on a multiplex qSAT assay performance on different dilutions of test and positive control samples based on crude fluorescence measurements. Using serial dilutions of a positive control to fit sigmoidal curves into a non-linear equation gives the antigen-specific quantifiable ranges used to choose the optimal sample dilution, increasing the sensitivity to assess the effect of different assay conditions on assay variability. These curves could also be applied to normalize day-to-day variability. We have shown that each antigen and sample combinations have an optimal sample dilution to assess variability, which depends on antigen immunogenicity and sample immunogenic profile. Consequently, when working with multidimensional data, crude fluorescence measurements are limited to find an effect on assay variability of different assay conditions, and this may drive to wrong conclusions. To overcome this difficulty we performed MAD by antigen and sample dilution for each condition [46].

The variability of the 16 combinations of conditions for all the positive control dilutions together and per antigen, showed that the combination with lower variability for most antigens was a unique coupling of beads with daily sample predilution and automatic plate washer. When ranking the MAD of the 16 combinations of conditions for all the positive control dilutions and all antigens together, the only difference between the combinations giving the lowest and the highest variability was the antigen-bead coupling (stock vs. several, respectively), showing that stock coupling was very important to reduce variability. However, we cannot discard the effect on variability of one condition on another.

The linear regression models showed that when assaying the positive control, stock coupling and sample incubation at 37°C resulted in less variability. Accordingly, variability increased if coupling was performed several times along the study and samples were incubated at 22°C compared to incubating at 37°C. Interestingly, the use of a unique coupling reduced the variability, no matter the temperature of sample incubation. Similarly, when assessing variability in the blanks we observed an increase of variability when stock coupling was not in the models, suggesting that stock coupling reduces variability of background signal. The procedure to wash plates did not affect the variability of the assay. However, in the analysis of combined conditions by the MAD, automatic washing seemed to reduce variability. Given the time and resources that automatic washing needs, and knowing that manual washing does not significantly add more variability, we consider acceptable to perform manual washing if this is the only option available.

Operator expertise did not affect assay variability, but the MFI quantification range was reduced when an apprentice performed the assay. Further studies assessing the operator day-to-day and inter-laboratory variability are needed to have more accurate reproducibility measurements.

Optimizing qSAT plate design, keeping the optimal number of replicates and dilutions, is crucial to ensure assay quality, being especially relevant in large immunological studies. Replicate measurements of the same sample provide random error estimates and direct estimates of variability. They also reduce the number of false negatives without increasing the number of false positives [47]. Our positive control data showed that some antigens presented less discrepancy between replicates in the first dilution (AMA-1, EBA-175, MSP-1_42_) and some others between replicates in the third dilution (VAR2CSA and SSP2). Thus, we cannot make any recommendation with regards to replicates of specific sample dilutions because robustness was antigen-specific. However, overall differences between replicates of test samples were minimal. In addition, to increase the sensitivity of a multiplex qSAT assay we have to ensure that sample MFIs fall in the linear part of the antigen-specific curve. Thus, in assays with multiplexed antigens and samples with different seroreactivities, having less replicates and more sample dilutions may increase the likelihood of MFIs falling in the linear part of the antigen-specific curve, increasing assay precision. Finally, we also showed that two blank replicates had the same ICCs estimates as three replicates, thus two blanks per plate would be sufficient.

Our study had as main limitation that the operator expertise was later included in the analysis, thus the fractional factorial design did not include this variable when the study was conceived. Hence, some of the models might lack power to detect true differences on assay variability.

## Conclusion

The qSAT is a robust assay to measure a broad range of antibody specificities against multiple antigens, giving reproducible measurements. Fractional factorial design allowed us to measure the effect of several conditions on assay variability, reducing the number of experiments and giving maximum sensitivity and accuracy. We showed that a single antigen-bead coupling for the whole study was the variable that most consistently reduced assay variability, being clearly advisable. In addition, whenever possible, prediluting samples at the beginning of the study, as well as washing plates automatically are recommended. Finally, in multiplex qSAT assays with antigens of different immunogenicities, adding more sample dilutions instead of replicates may increase the likelihood of MFIs falling in the linear part of the antigen-specific curve thus increasing assay sensitivity and precision.

## Supporting information

S1 Table Fractional factorial design of the qSAT experiments. The assay conditions analyzed were coupling of the antigens to beads (stock vs. several), sample predilution (stock, vs. daily), temperature of incubation of samples with antigen-beads (22°C vs. 37°C), plate washing (manual vs. automatic) and operator expertise (expert vs. apprentice).
(XLSX)

S2 Table Study dataset. Data file with the variables described as follows: plate; type, multiplex (mp) or singleplex (sp); antigen, the antigen analysed; Sample Type, Blank, Positive (positive controls), Standard (singleplex) or Subject; Sample Dil, number of sample dilution for each sample type; well, well on each plate; Dilution, dilution factor; Bead, coupling of the bead (stock vs. several); Predilution of the sample (stock vs. daily); Temperature of sample-beads incubation (22°C vs. 37°C), Washing (automatic vs. manual) and Condition, all condition together; mfi, median fluorescence intensity; and log10_mfi.
(XLSX)

S1 Fig Example of plate design. Each plate included nine subject samples in 4 serial dilutions with duplicates in alternate positions (M1-M9, in blue), a positive control in 8 serial dilutions, with replicates in alternate positions (PosCtrl, in red), and three blanks with multiplex antigen-coupled beads (Blanks, in yellow).
(PDF)

S2 Fig Antigen-specific log_10_MFI levels of positive control serial dilutions for all assay conditions and antigens analyzed. Spaghetti plots represent examples of positive control serial dilution MFIs against different antigens and in different assay conditions: Antigen-bead coupling (stock vs. several), sample predilution (stock vs. daily), temperature of sample-beads incubation (22°C vs. 37°C), plate washing (automatic vs. manual) and operator expertise (expert vs. apprentice). Grey lines correspond to data from each plate and black lines are loess fitted.
(PDF)

S3 Fig Median absolute deviation (MAD) of log_10_MFI of positive control serial dilutions for each assay condition and antigen. Conditions analyzed were: Antigen-bead coupling (stock vs. several), sample predilution (stock vs. daily), temperature of incubation of samples with antigen-beads (22°C vs. 37°C), plate washing (automatic vs. manual) and operator expertise (experienced vs. apprentice).
(PDF)

S4 Fig Bland-Altman plots representing the differences of positive control replicates against its mean for all antigens. Dashed blue lines show the 95% confidence interval of the differences.
(PDF)
