# Supplementary material for: Analysis of factors affecting the variability of a quantitative suspension bead array assay measuring IgG to multiple Plasmodium antigens
Source: PLoS One. 2018 Jul 2;13(7):e0199278. doi: 10.1371/journal.pone.0199278 (PMC6028107; doi:10.1371/journal.pone.0199278)
Supplement: S1 Fig — Each plate included nine subject samples in 4 serial dilutions with duplicates in alternate positions (M1-M9, in blue), a positive control in 8 serial dilutions, with replicates in alternate positions (PosCtrl, in red), and three blanks with multiplex antigen-coupled beads (Blanks, in yellow). (PDF) [file pone.0199278.s003.pdf]

	1	2	3	4	5	6	7	8	9	10	11	12
A	Std_01A	Std_02A	Std_03B	Std_05A	Std_06A	Std_07B	Std_09A	Std_10A	Std_11B	Std_13A	Std_14A	Std_15B
B	Std_01B	Std_03A	Std_04A	Std_05B	Std_07A	Std_08A	Std_09B	Std_11A	Std_12A	Std_13B	Std_15A	Std_16A
C	M1_1A	M1_1B	M1_2A	M1_3A	M1_3B	M1_4A	M5_1A	M5_2A	M5_2B	M5_3A	M5_4A	M5_4B
D	M2_1A	M2_1B	M2_2A	M2_3A	M2_3B	M2_4A	M6_1A	M6_2A	M6_2B	M6_3A	M6_4A	M6_4B
E	M3_1A	M3_1B	M3_2A	M3_3A	M3_3B	M3_4A	M7_1A	M7_2A	M7_2B	M7_3A	M7_4A	M7_4B
F	M4_1A	M4_1B	M4_2A	M4_3A	M4_3B	M4_4A	M8_1A	M8_2A	M8_2B	M8_3A	M8_4A	M8_4B
G	Blank_1	Blank_2	Blank_3	PosCtrl_02A	PosCtrl_04A	PosCtrl_06A	M9_1A	M9_2A	M9_2B	M9_3A	M9_4A	M9_4B
H	PosCtrl_01A	PosCtrl_01B	PosCtrl_03A	PosCtrl_03B	PosCtrl_05A	PosCtrl_05B	PosCtrl_07A	PosCtrl_07B	PosCtrl_08A			

Singleplex Standard Curve (Std)
Subject samples (M1-M9)
Blanks
Positive Control (PosCtrl)
