# Supplementary material for: Analysis of factors affecting the variability of a quantitative suspension bead array assay measuring IgG to multiple Plasmodium antigens
Source: PLoS One. 2018 Jul 2;13(7):e0199278. doi: 10.1371/journal.pone.0199278 (PMC6028107; doi:10.1371/journal.pone.0199278)
Supplement: S2 Fig — Spaghetti plots represent examples of positive control serial dilution MFIs against different antigens and in different assay conditions: Antigen-bead coupling (stock vs. several), sample predilution (stock vs. daily), temperature of sample-beads incubation (22°C vs. 37°C), plate washing (automatic vs. manual) and operator expertise (expert vs. apprentice). Grey lines correspond to data from each plate and black lines are loess fitted. (PDF) [file pone.0199278.s004.pdf]

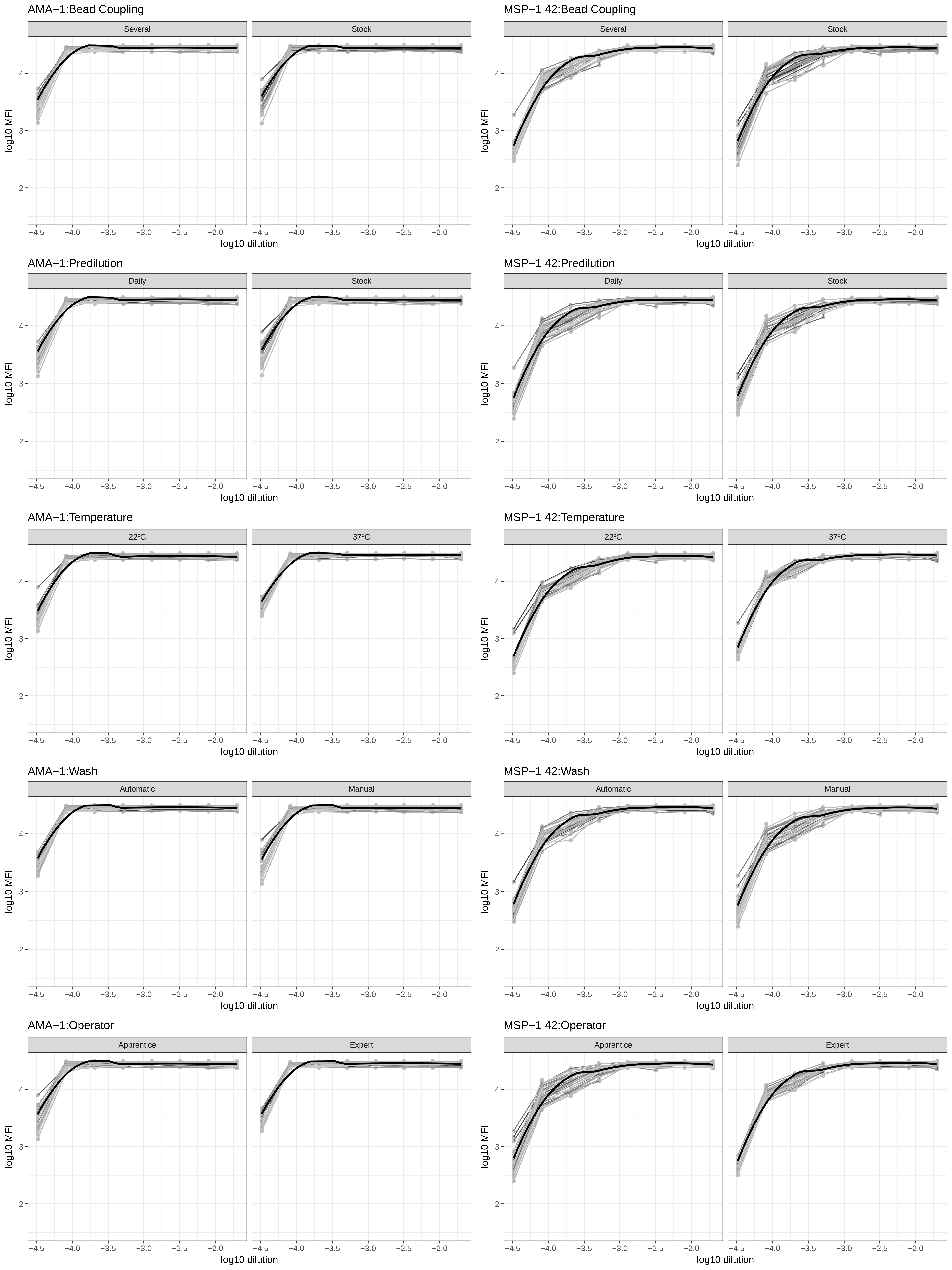
AMA−1:Bead Coupling
Several
Stock
MSP−1 42:Bead Coupling
Several
Stock
AMA−1:Predilution
Daily
Stock
MSP−1 42:Predilution
Daily
Stock
AMA−1:Temperature
22ºC
37ºC
MSP−1 42:Temperature
22ºC
37ºC
AMA−1:Wash
Automatic
Manual
MSP−1 42:Wash
Automatic
Manual
AMA−1:Operator
Apprentice
Expert
MSP−1 42:Operator
Apprentice
Expert
log10 MFI
log10 dilution
2
3
4
−4.5
−4.0
−3.5
−3.0
−2.5
−2.0

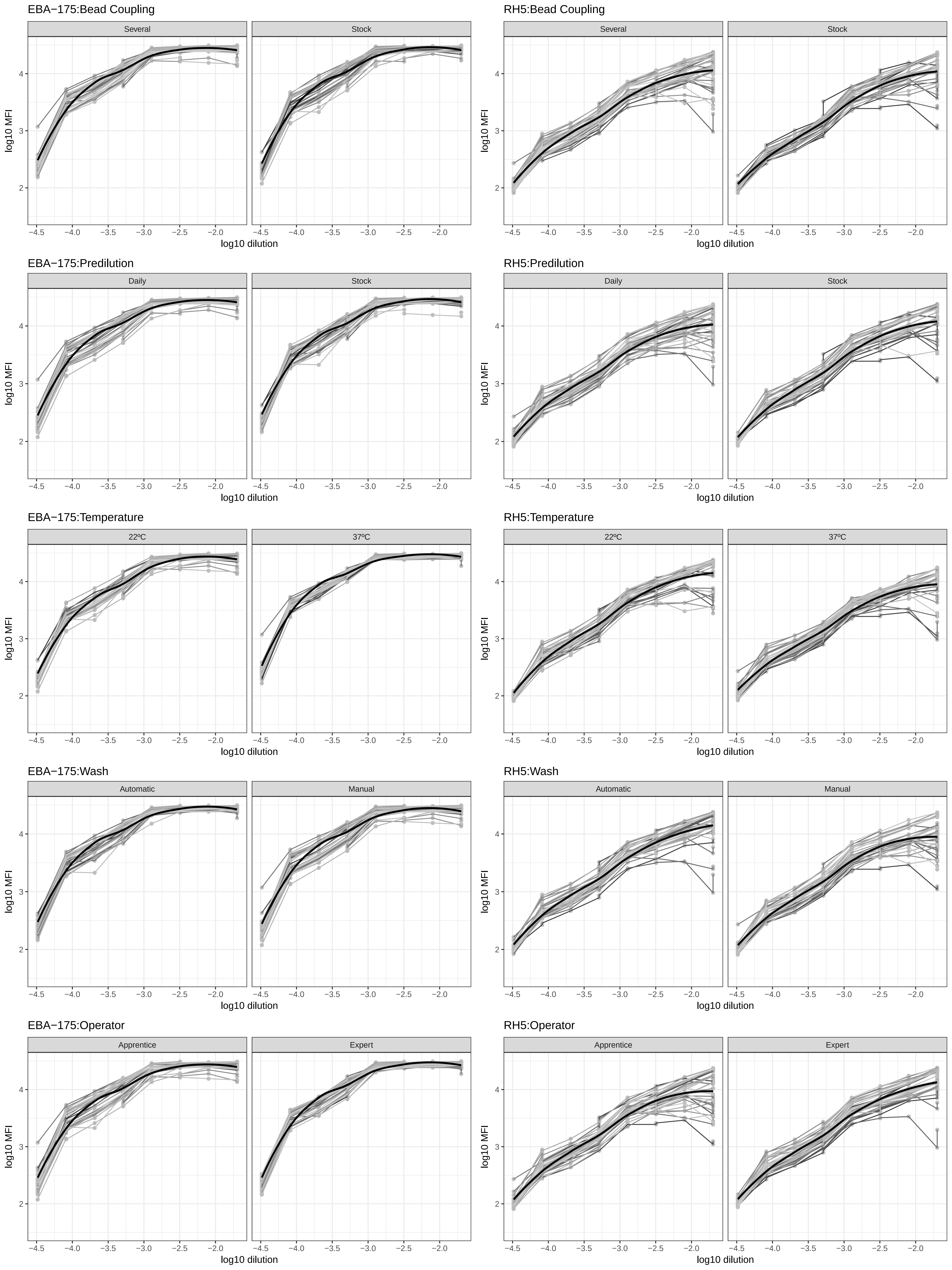
EBA−175:Bead Coupling
Several
Stock
RH5:Bead Coupling
Several
Stock
EBA−175:Predilution
Daily
Stock
RH5:Predilution
Daily
Stock
EBA−175:Temperature
22ºC
37ºC
RH5:Temperature
22ºC
37ºC
EBA−175:Wash
Automatic
Manual
RH5:Wash
Automatic
Manual
EBA−175:Operator
Apprentice
Expert
RH5:Operator
Apprentice
Expert
log10 MFI
log10 dilution
-4.5
-4.0
-3.5
-3.0
-2.5
-2.0
2
3
4

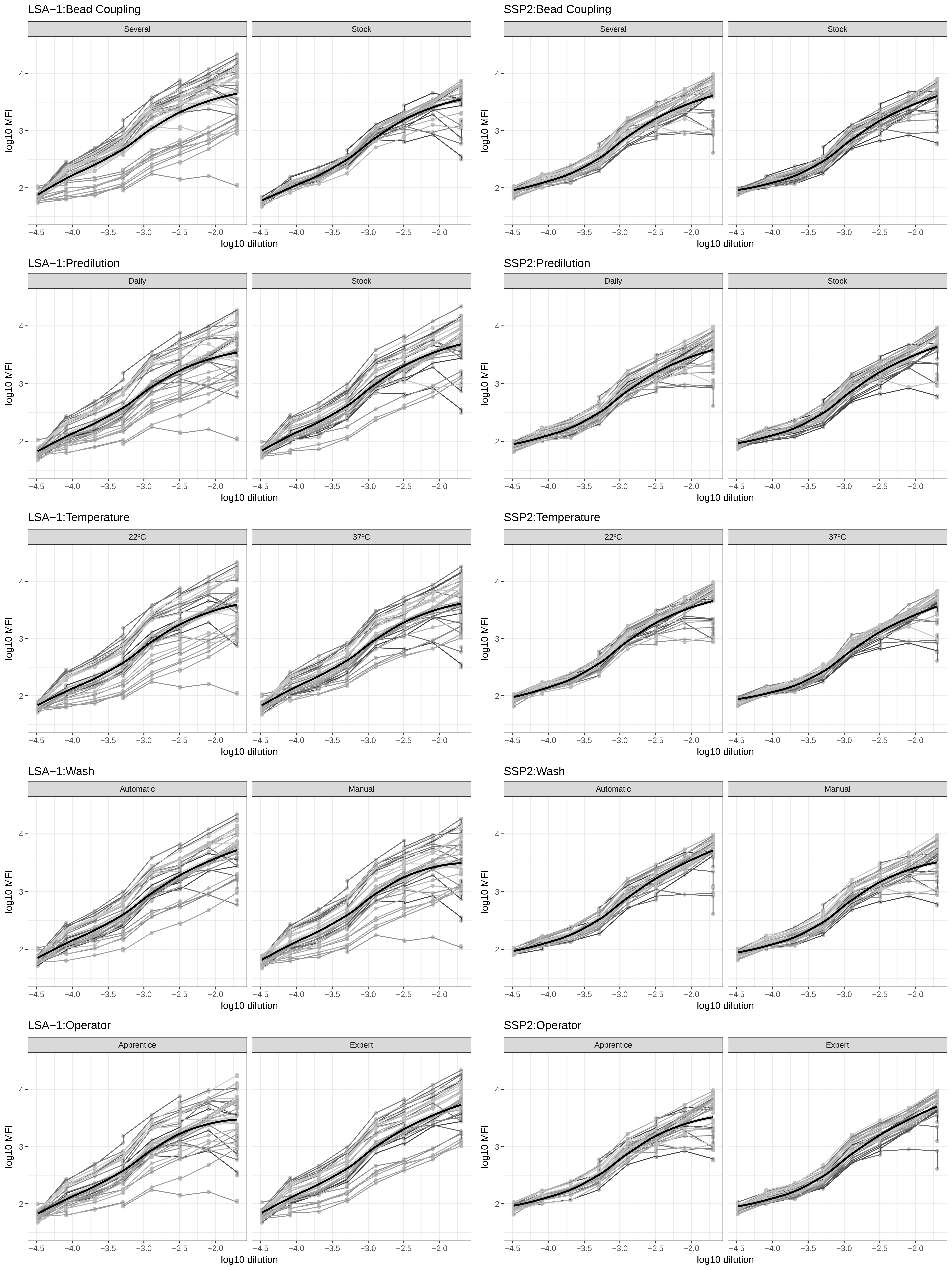
LSA−1:Bead Coupling
Several
Stock
SSP2:Bead Coupling
Several
Stock
LSA−1:Predilution
Daily
Stock
SSP2:Predilution
Daily
Stock
LSA−1:Temperature
22ºC
37ºC
SSP2:Temperature
22ºC
37ºC
LSA−1:Wash
Automatic
Manual
SSP2:Wash
Automatic
Manual
LSA−1:Operator
Apprentice
Expert
SSP2:Operator
Apprentice
Expert
log10 MFI
log10 dilution
−4.5
−4.0
−3.5
−3.0
−2.5
−2.0
2
3
4

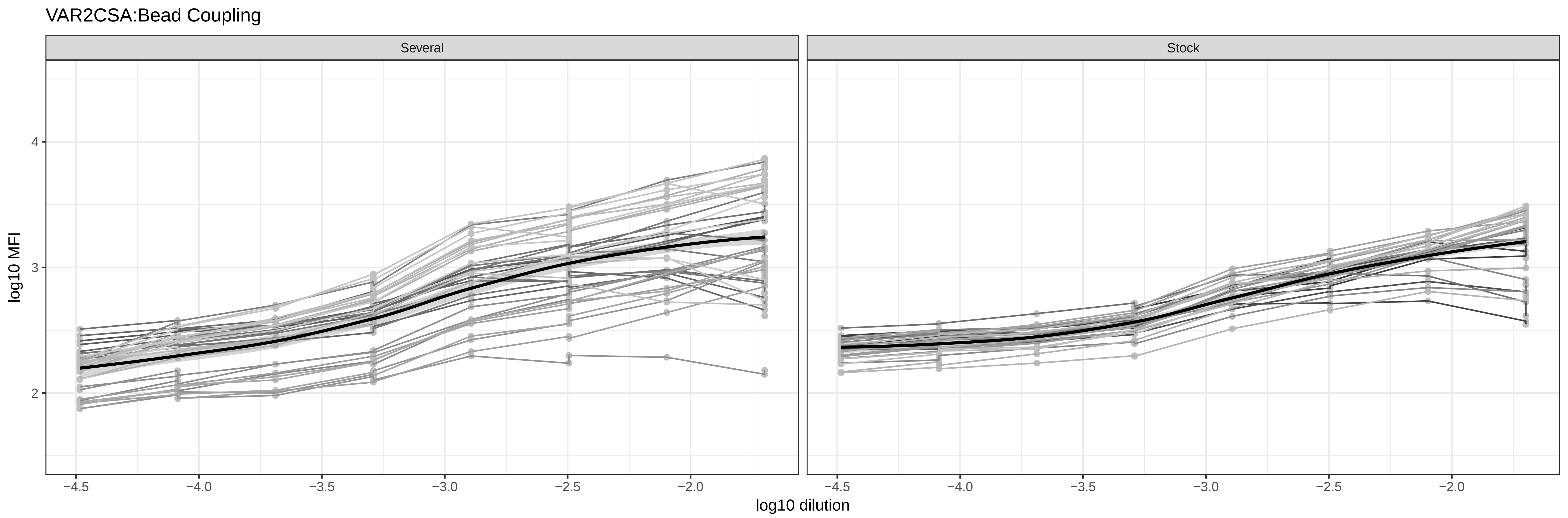
VAR2CSA:Bead Coupling
Several
Stock
log10 MFI
4
3
2
−4.5
−4.0
−3.5
−3.0
−2.5
−2.0
log10 dilution

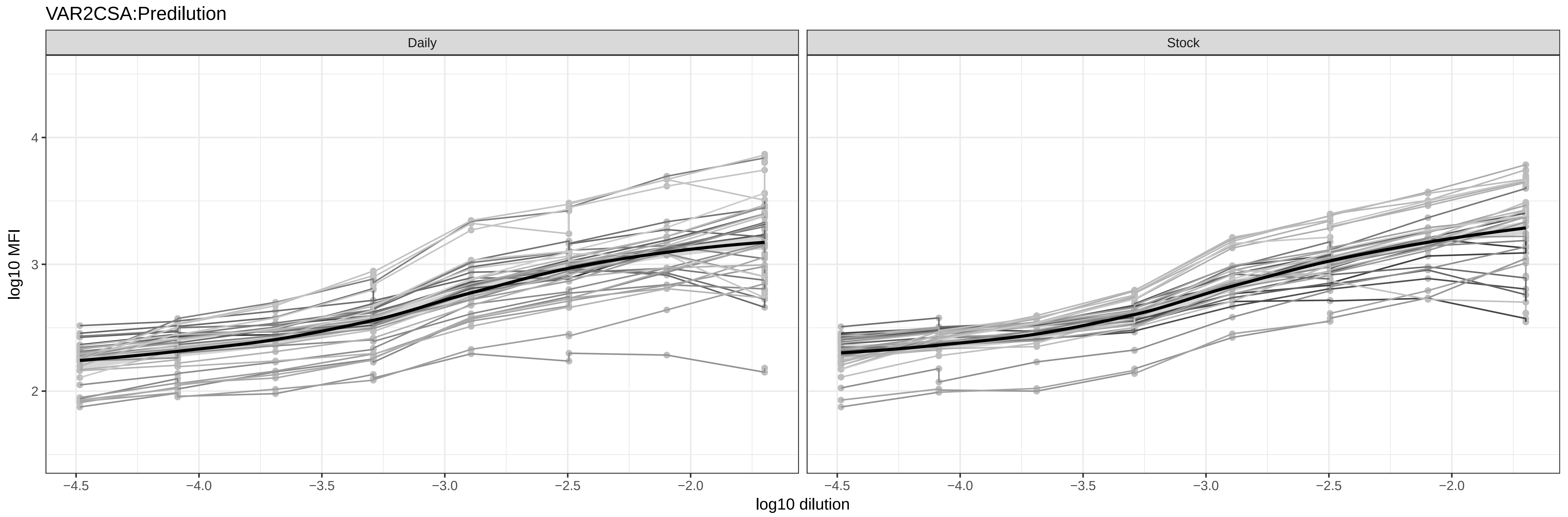
VAR2CSA:Predilution
Daily
Stock
log10 MFI
4
3
2
−4.5
−4.0
−3.5
−3.0
−2.5
−2.0
log10 dilution

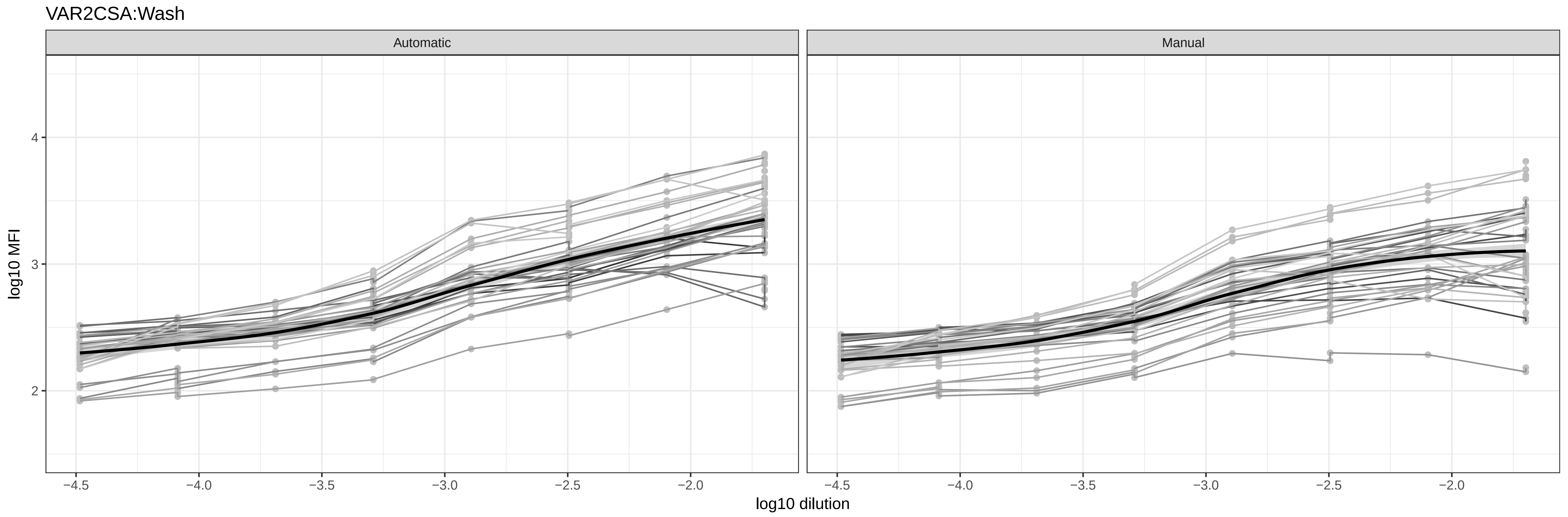
VAR2CSA:Wash
Automatic
Manual
log10 MFI
4
3
2
−4.5
−4.0
−3.5
−3.0
−2.5
−2.0
log10 dilution

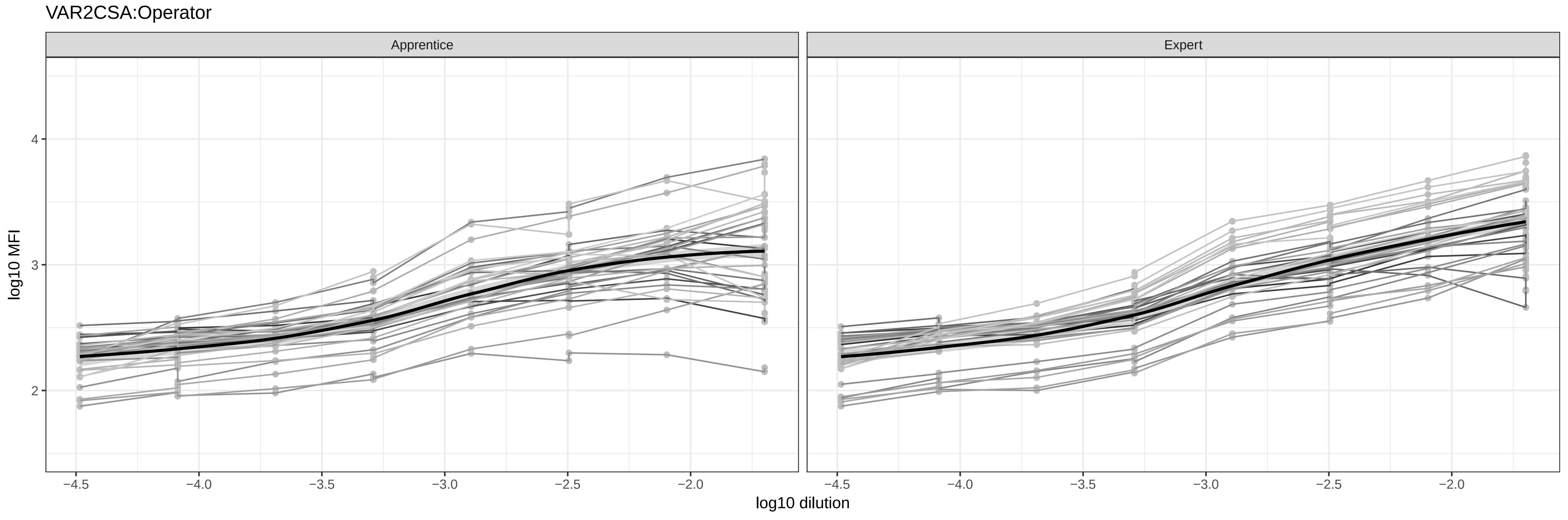
VAR2CSA:Operator
Apprentice
Expert
log10 MFI
4
3
2
−4.5
−4.0
−3.5
−3.0
−2.5
−2.0
log10 dilution
