# Supplementary figures and images for: Analysis of factors affecting the variability of a quantitative suspension bead array assay measuring IgG to multiple Plasmodium antigens
Source: PLoS One. 2018 Jul 2;13(7):e0199278. doi: 10.1371/journal.pone.0199278 (PMC6028107; doi:10.1371/journal.pone.0199278)

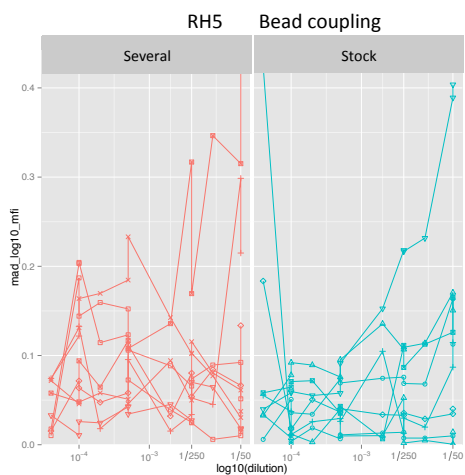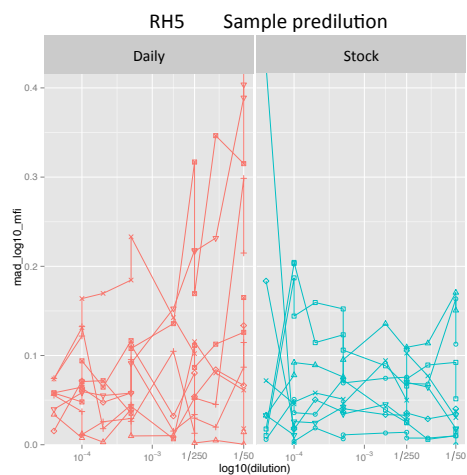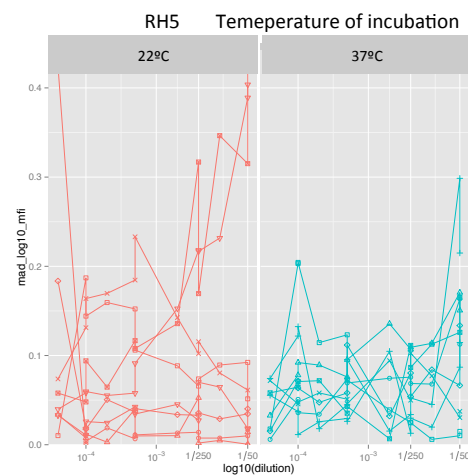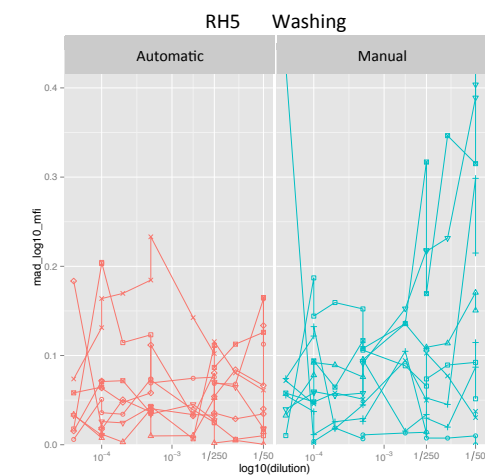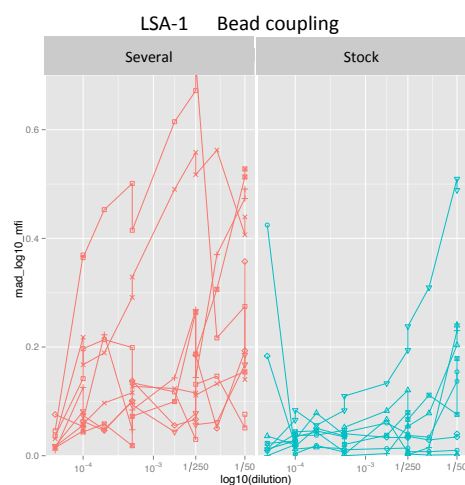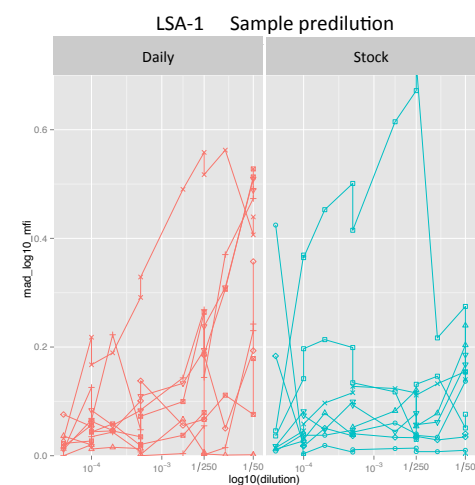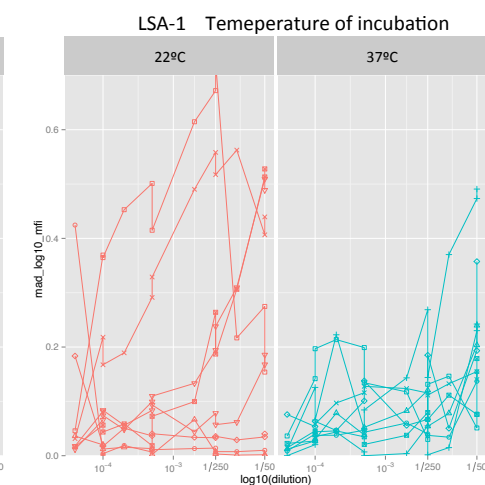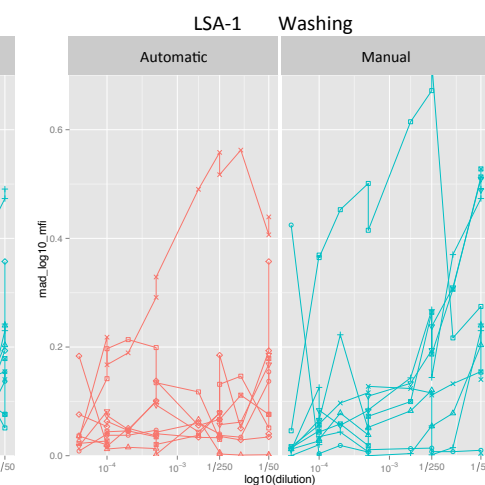

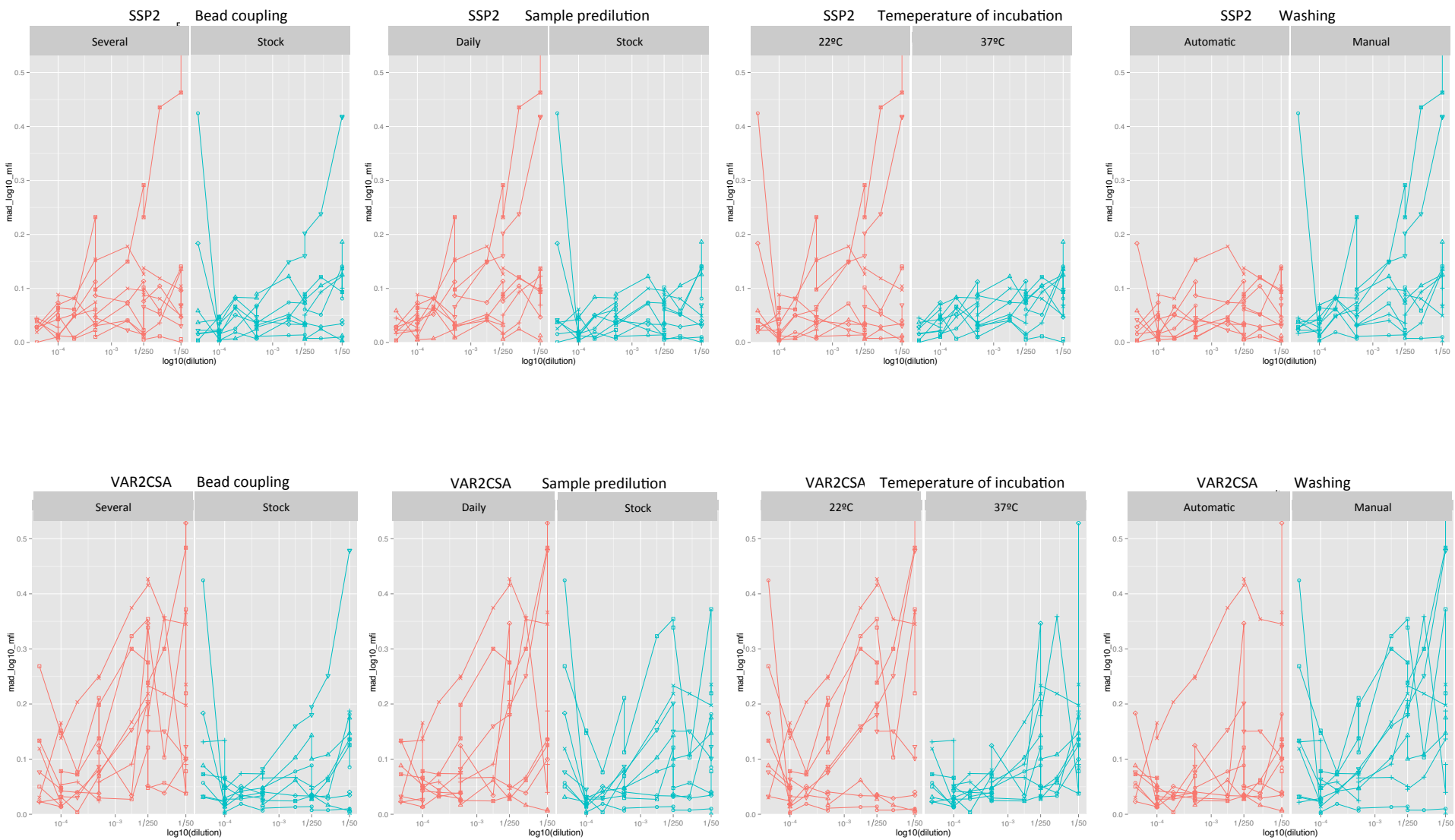

Supplement: S3 Fig — Conditions analyzed were: Antigen-bead coupling (stock vs. several), sample predilution (stock vs. daily), temperature of incubation of samples with antigen-beads (22°C vs. 37°C), plate washing (automatic vs. manual) and operator expertise (experienced vs. apprentice). (PDF) [file pone.0199278.s005.pdf]
