# Supplementary material for: Analysis of factors affecting the variability of a quantitative suspension bead array assay measuring IgG to multiple Plasmodium antigens
Source: PLoS One. 2018 Jul 2;13(7):e0199278. doi: 10.1371/journal.pone.0199278 (PMC6028107; doi:10.1371/journal.pone.0199278)
Supplement: S4 Fig — Dashed blue lines show the 95% confidence interval of the differences. (PDF) [file pone.0199278.s006.pdf]

Positive control dilution: AMA-1

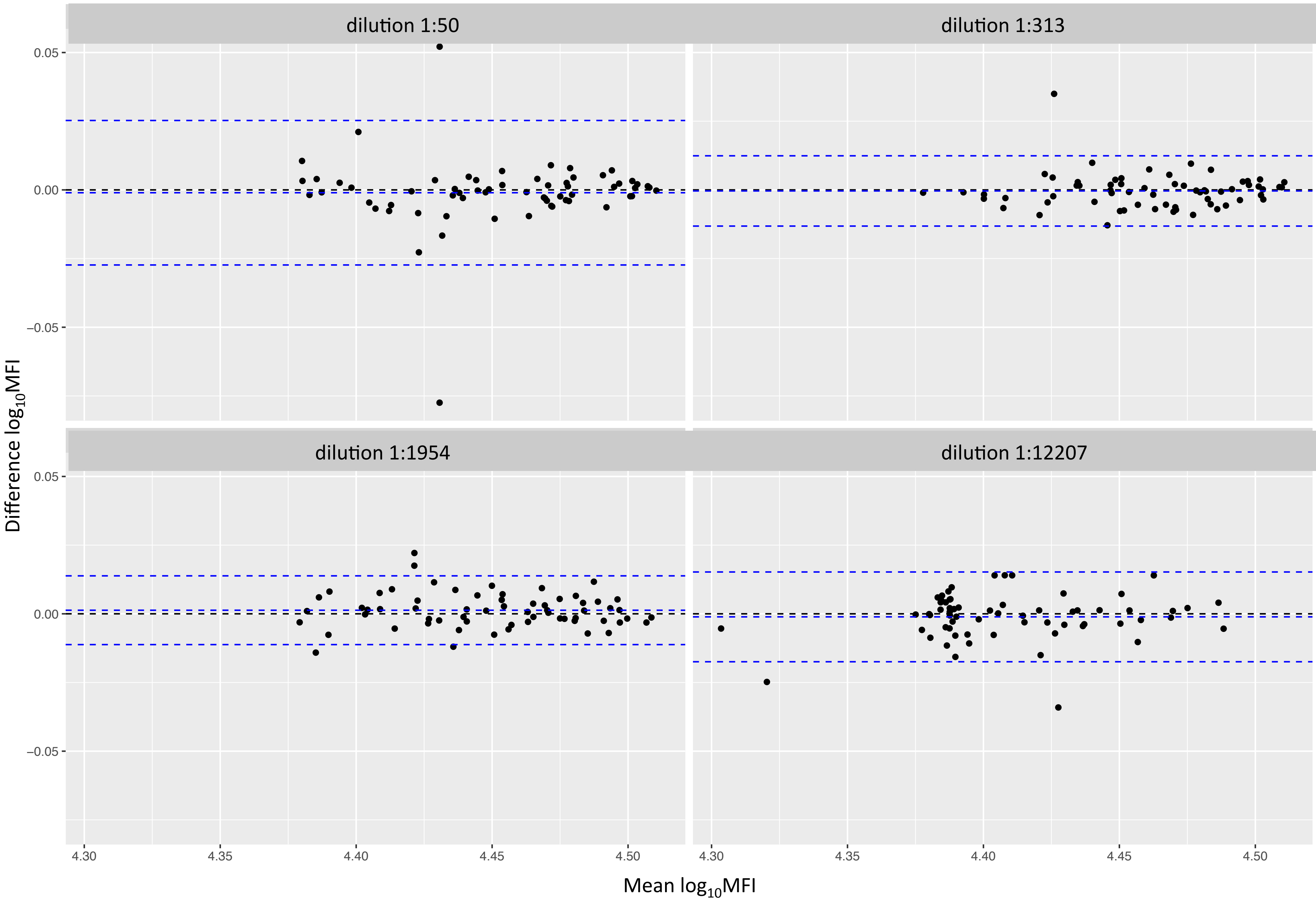

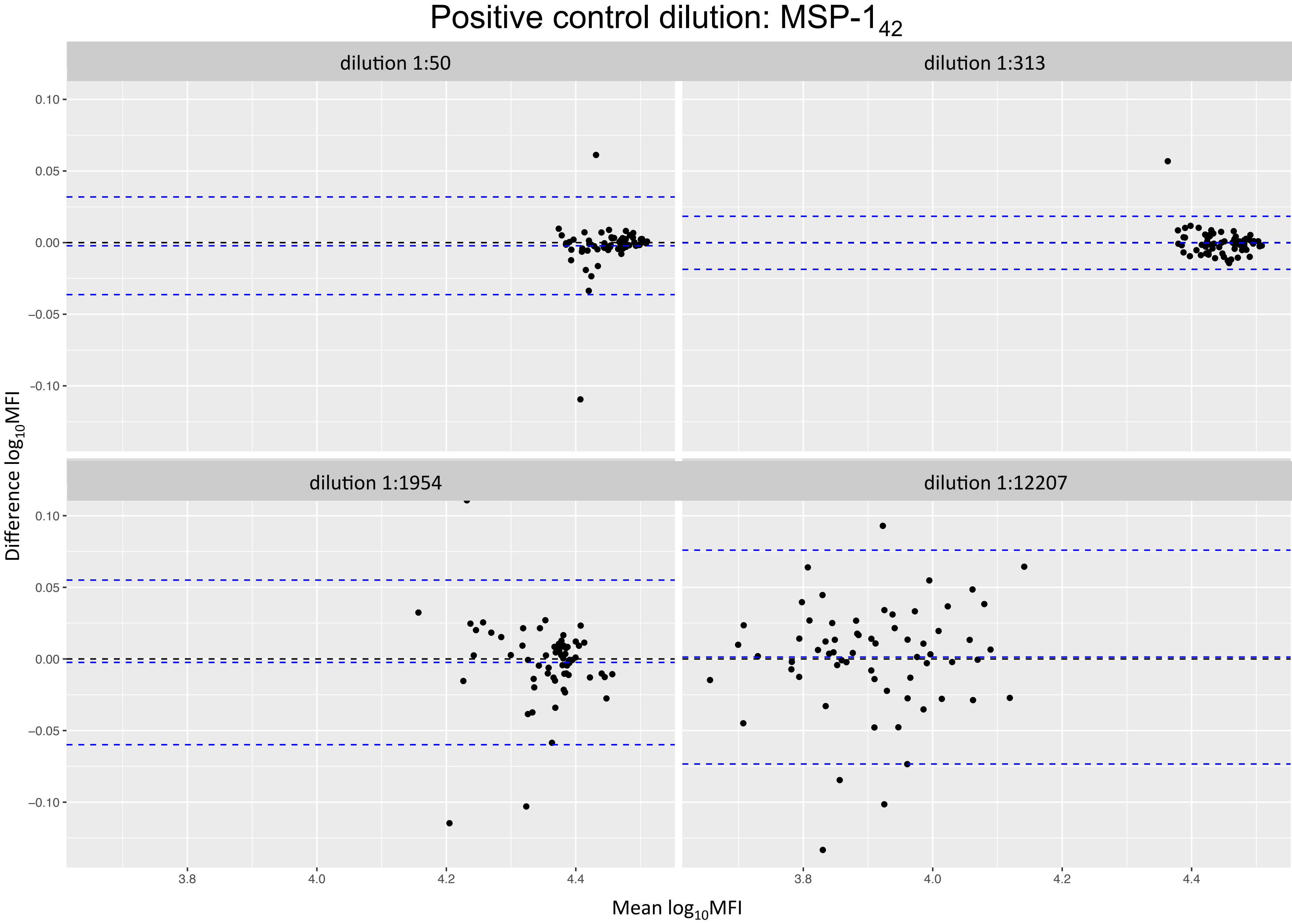
Positive control dilution: MSP-1$_{42}$
dilution 1:50
dilution 1:313
dilution 1:1954
dilution 1:12207
Difference log$_{10}$MFI
Mean log$_{10}$MFI
0.10
0.05
0.00
−0.05
−0.10
3.8
4.0
4.2
4.4

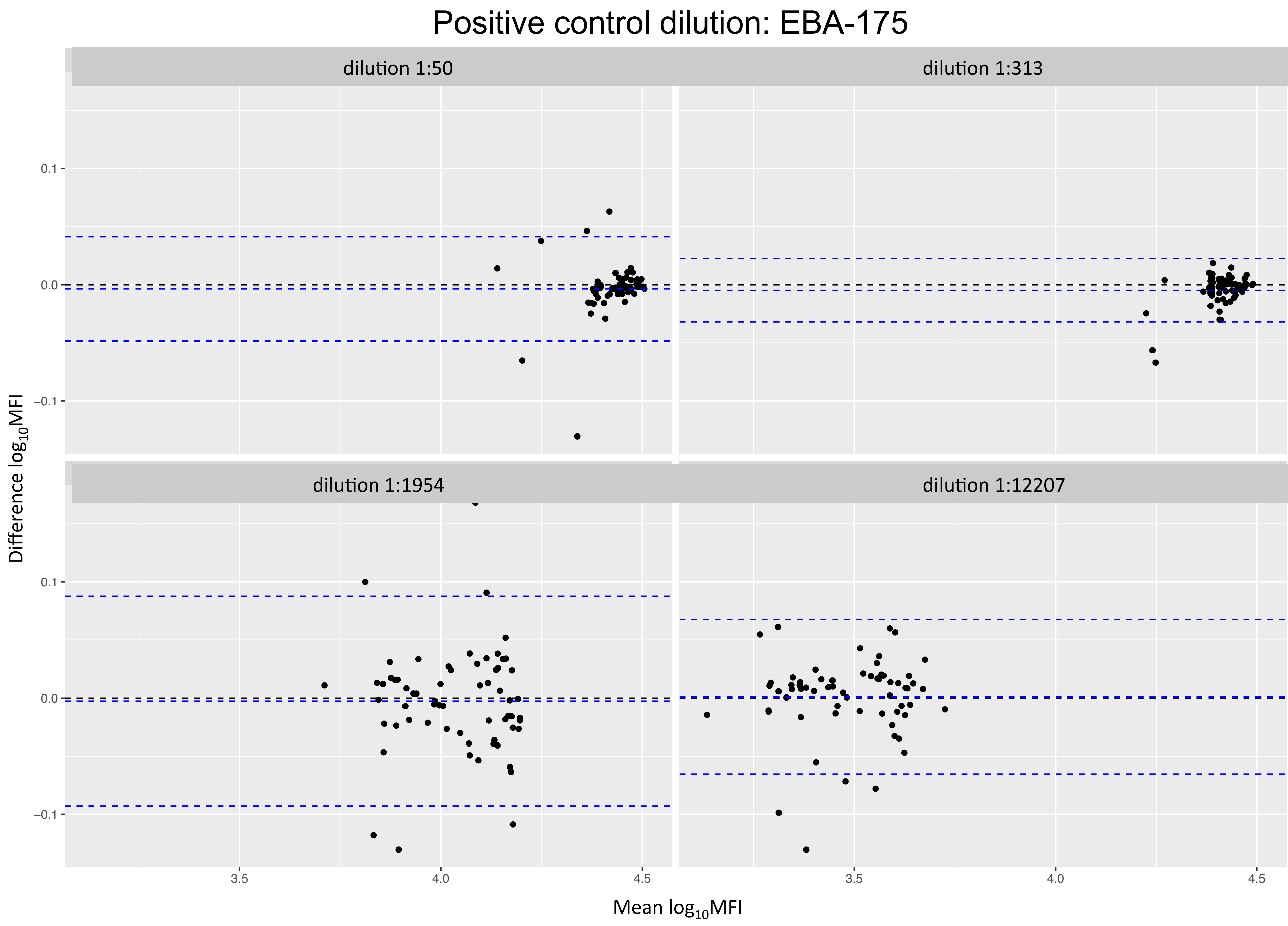

Positive control dilution: EBA-175
dilution 1:50
dilution 1:313
dilution 1:1954
dilution 1:12207
0.1
0.0
−0.1
3.5
4.0
4.5
Difference log10MFI
Mean log10MFI

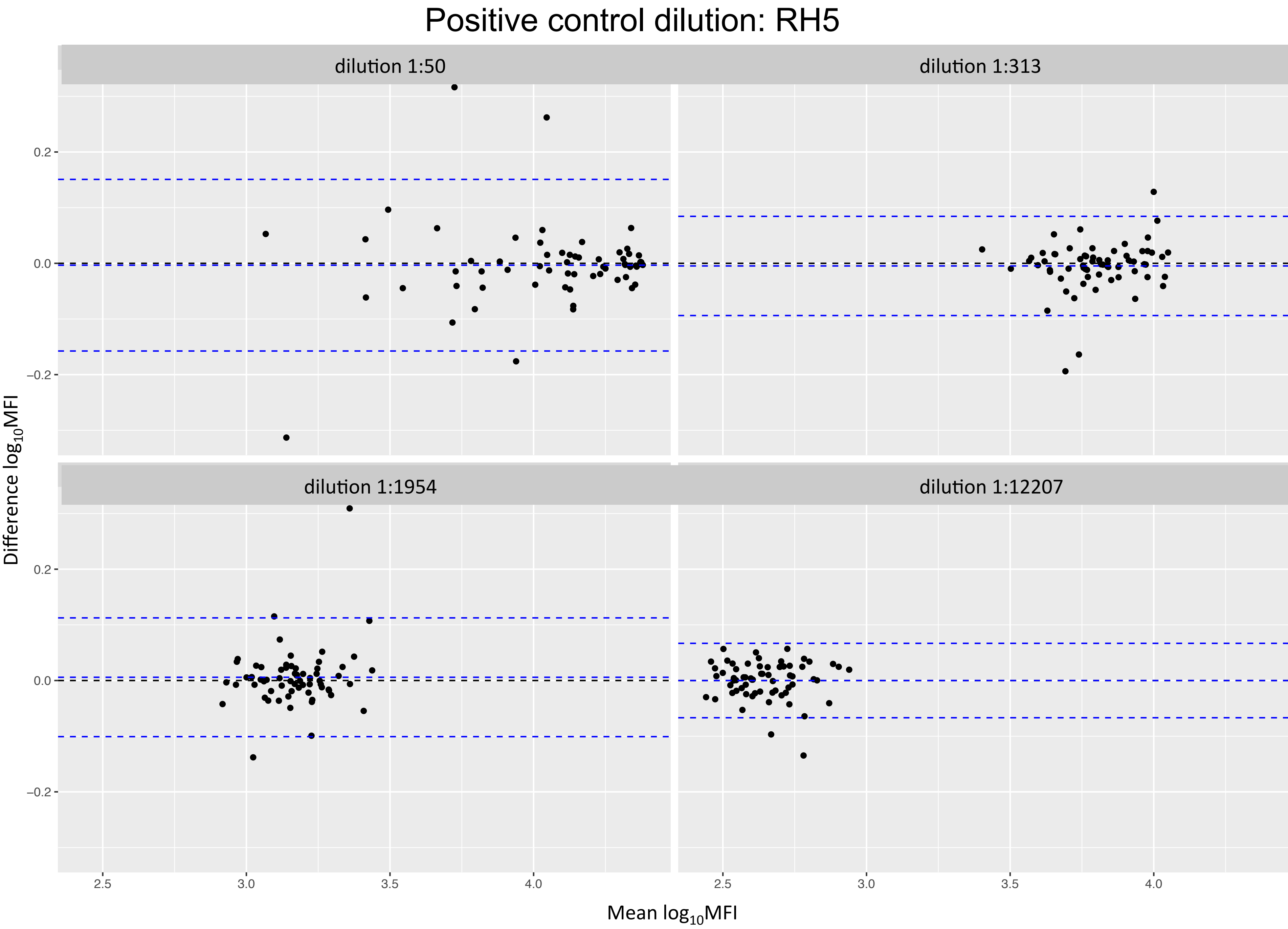

Positive control dilution: RH5
dilution 1:50
dilution 1:313
dilution 1:1954
dilution 1:12207
Difference $\log_{10}$MFI
Mean $\log_{10}$MFI
0.2
0.0
−0.2
2.5
3.0
3.5
4.0

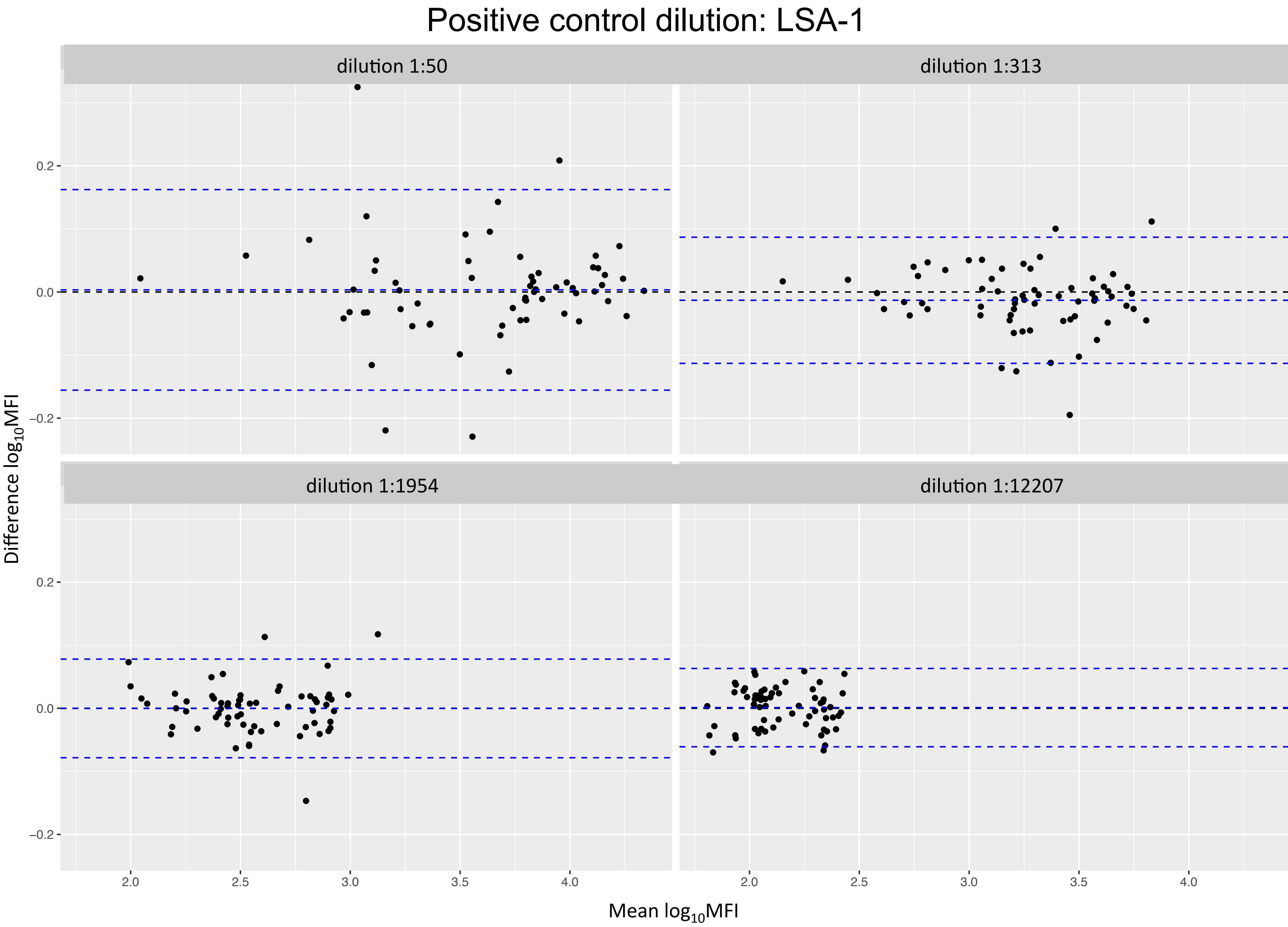

Positive control dilution: LSA-1
dilution 1:50
dilution 1:313
dilution 1:1954
dilution 1:12207
0.2
0.0
−0.2
2.0
2.5
3.0
3.5
4.0
Difference log10MFI
Mean log10MFI

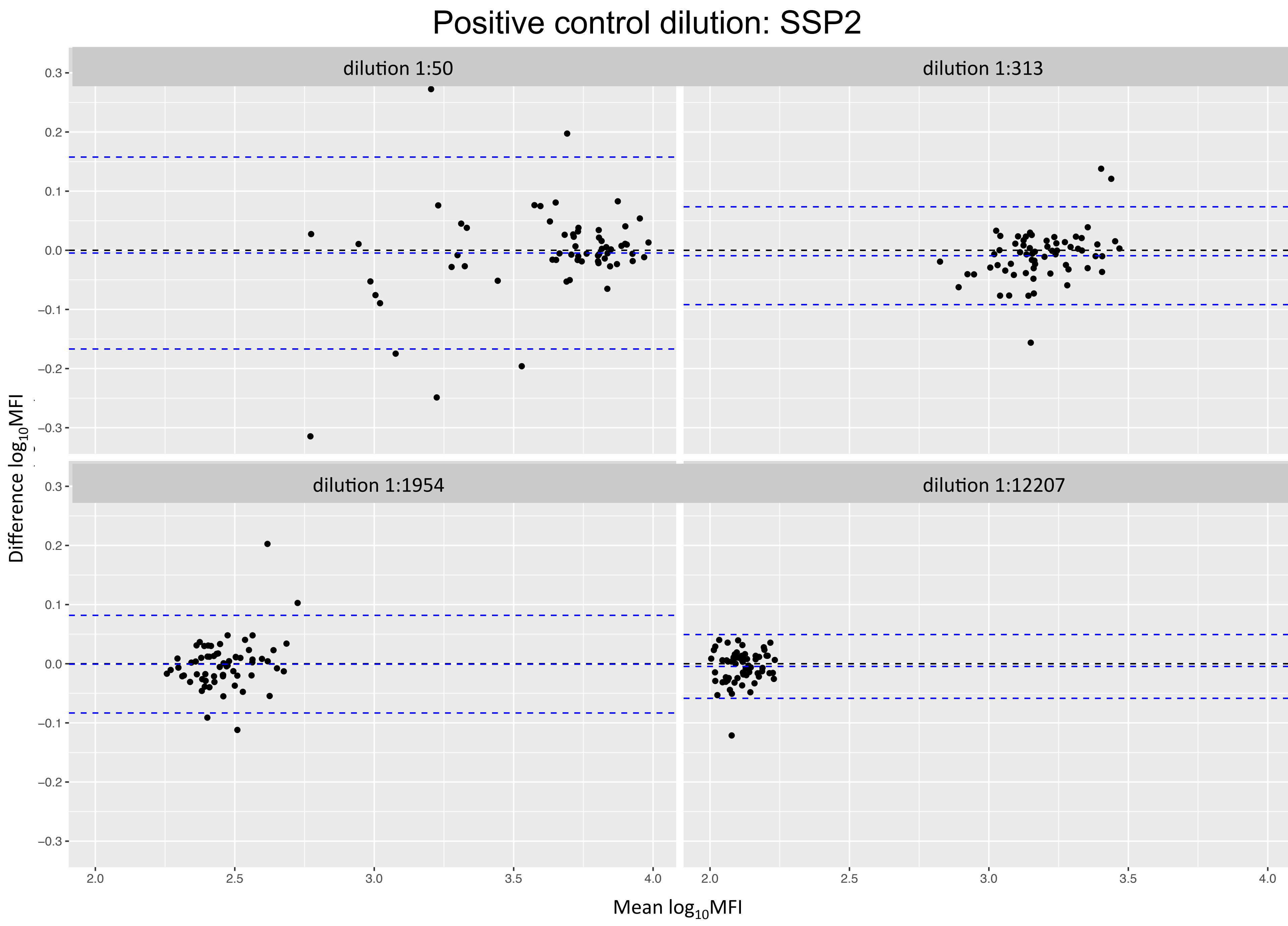
Positive control dilution: SSP2
dilution 1:50
dilution 1:313
dilution 1:1954
dilution 1:12207
Difference log_{10}MFI
Mean log_{10}MFI
0.3
0.2
0.1
0.0
−0.1
−0.2
−0.3
2.0
2.5
3.0
3.5
4.0

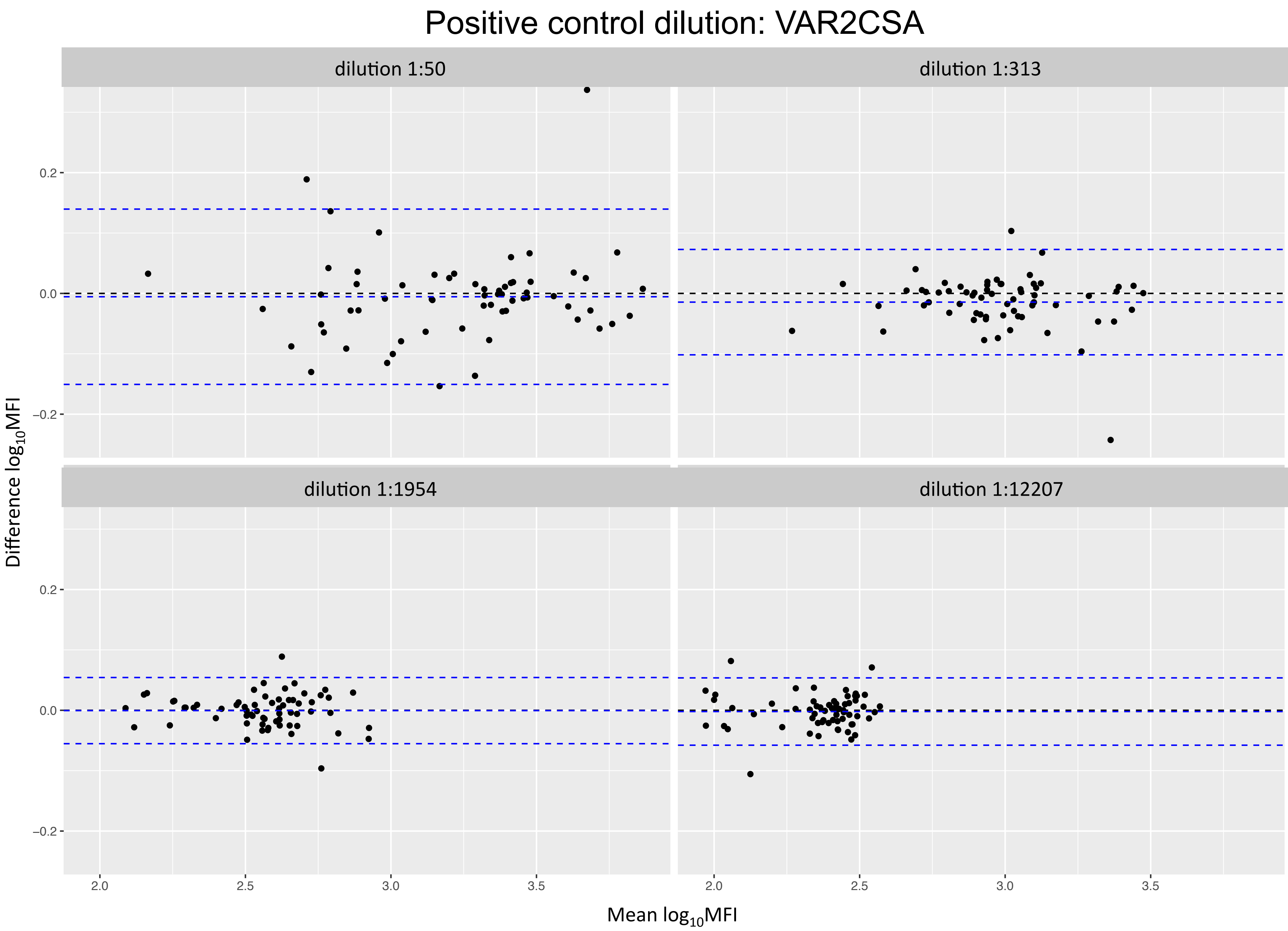
Positive control dilution: VAR2CSA
dilution 1:50
dilution 1:313
dilution 1:1954
dilution 1:12207
Difference log10MFI
Mean log10MFI
0.2
0.0
−0.2
2.0
2.5
3.0
3.5
